# Supplementary material for: ENOblock synergizes with colistin to treat Acinetobacter baumannii infections
Source: EMBO Mol Med. 2025 Oct 31;17(12):3496–524. doi: 10.1038/s44321-025-00331-2 (PMC12686454; doi:10.1038/s44321-025-00331-2)
Supplement: Supplementary file 1 — Appendix [file 44321_2025_331_MOESM1_ESM.pdf]

Appendix for “ENOblock synergizes with colistin to treat *Acinetobacter baumannii* infections”

Table of Contents

1. **Appendix Figure S1 …………………………………………..page 2**
2. **Appendix Figure S2 …………………………………………..page 3**
3. **Appendix Figure S3 …………………………………………..page 4**
4. **Appendix Figure S4 …………………………………………..page 5**
5. **Appendix Table S1 …………………………………………..page 6**
6. **Appendix Table S2 …………………………………………..page 7**
7. **Appendix Table S3 …………………………………………..page 8**
8. **Appendix Table S4 …………………………………………..page 9**
9. **Appendix Table S5 …………………………………………..page 10**

Appendix Figure S1

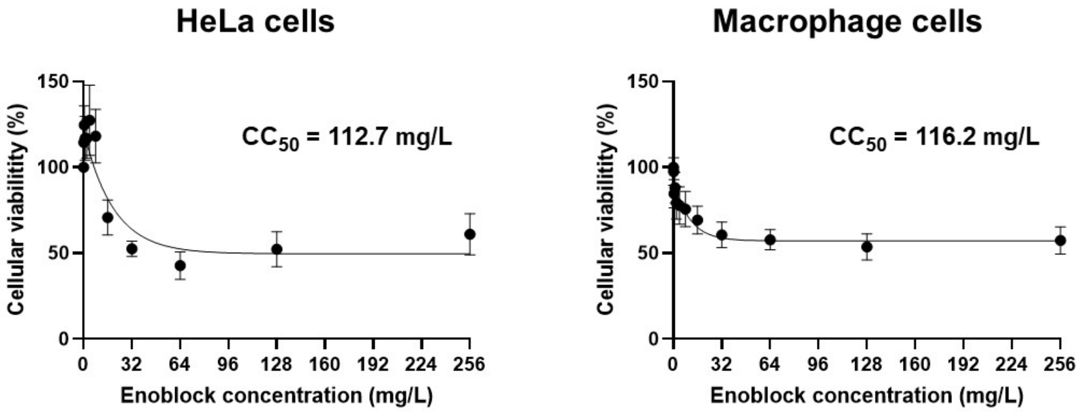

Appendix Figure S1. Cellular viability of epithelial and macrophage cells in presence of different concentration of ENOblock.

Appendix Figure S2

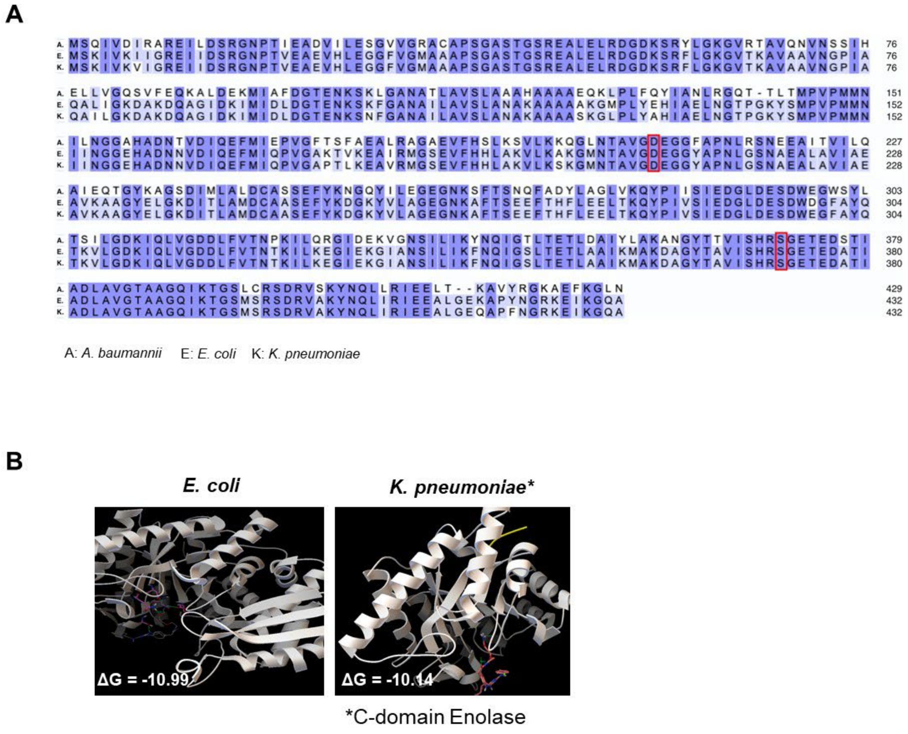

Appendix Figure S2. ENOblock acts on *E. coli and K. pneumoniae* through the inhibition of enolase. (**A**) The enolase sequences of *A. baumannii*, *E. coli* and *K. pneumoniae*. (**B**) Structural models generated by docking of ENOblock into *E. coli* and *K. pneumoniae* enolase. ENOblock is displayed as sticks. ΔG: Glide score.

Appendix Figure S3

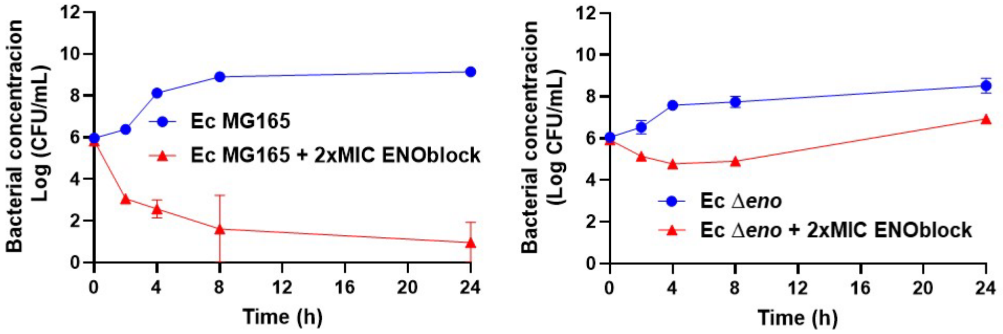

Appendix Figure S3. ENOblock acts on *E. coli* through the inhibition of enolase. Time-kill curves of *E. coli* Ec MG165 ⁺ and Ec Δ*eno* strains in the presence of 2xMIC ENOblock for 24 hours.

Appendix Figure S4

```
Homo sapiens   m s i e k i w a r e i l d s r g n p t v e v d l y t a k g l f r a a v p s g a s t g i y e a l e l r d g d k q r y l g k g v l k a v d h i n s t i a p a 76
A. baumannii   m s i e k i w a r e i l d s r g n p t v e v d l y t a k g l f r a a v p s g a s t g i y e a l e l r d g d k q r y l g k g v l k a v d h i n s t i a p a 76
G. mellonella  m p i k s i k a r q i f d s r g n p t v e v d l v t e l g l f r a a v p s g a s t g v h e a l e l r d n i k n q y h g k g v l t a i k n i n e i i a p a 76

Homo sapiens   l i s s g l s v v e q e k l d n l m l e l d g t e n k s k f g a n a i l g v s l a v c k a g a a e r e l p l y r h i a q l a g n s d l i l p v p a f n v 152
A. baumannii   l i s s g l s v v e q e k l d n l m l e l d g t e n k s k f g a n a i l g v s l a v c k a g a a e r e l p l y r h i a q l a g n s d l i l p v p a f n v 152
G. mellonella  l l k q n i d v t q q n e i d q f m i s l d g t e n k s k l g a n a i l g v s l a v a k a g a a s k g v p l y k h l a d l s g n n n i v l p v p a f n v 152

Homo sapiens   i n g - s h a g n k l a m q e f m i l p v g a e s f r d a m r l g a e v y h t l k g v i k d k y g k d a t n v g d e g g f a p n i l e n s e a l e l v k 227
A. baumannii   i n g - s h a g n k l a m q e f m i l p v g a e s f r d a m r l g a e v y h t l k g v i k d k y g k d a t n v g d e g g f a p n i l e n s e a l e l v k 227
G. mellonella  i n g g s h a g n k l a m q e f m i l p t g a t s f s e a m r m g s e v y h y l k k i i k e k f g l d s t a v g d e g g f a p n i l n n k d a l f l i q 228

Homo sapiens   e a i d k a g y t e k i v i g m d v a a s e f y r d g k y d l d f k s p t - d p s r y i t g d q l g a l y q d f v r d y p v v s i e d p f d q d d w a a 302
A. baumannii   e a i d k a g y t e k i v i g m d v a a s e f y r d g k y d l d f k s p t - d p s r y i t g d q l g a l y q d f v r d y p v v s i e d p f d q d d w a a 302
G. mellonella  d a i q q a g y t g k i e i g m d v a a s e f y k n g t y d l d f k n p k s n p a d y l p s d k l a d l y l e f i k e f p l v s i e d p f d q d d w a a 304

Homo sapiens   w s k f t a n v g i q i v g d d l t v t n p k r i e r a v e e k a c n c l l l k v n q i g s v t e a i q a c k l a q e n g w g v m v s h r s g e t e d t 378
A. baumannii   w s k f t a n v g i q i v g d d l t v t n p k r i e r a v e e k a c n c l l l k v n q i g s v t e a i q a c k l a q e n g w g v m v s h r s g e t e d t 378
G. mellonella  w s g l t s r t p i q i v g d d l t v t n p k r i a t a v e k k a c n c l l l k v n q i g s v t e s i q a h l l a k k n g w g t m v s h r s g e t e d t 380

Homo sapiens   f i a d l v v g l c t g q i k t g a p c r s e r l a k y n q l m r i e e e l g d e a r f a g h n f r n p s v l 433
A. baumannii   f i a d l v v g l c t g q i k t g a p c r s e r l a k y n q l m r i e e e l g d e a r f a g h n f r n p s v l 433
G. mellonella  f i a d l v v g l s t g q i k t g a p c r s e r l a k y n q i l r i e e e l g a a a k y a g k n f r r p v - - 433
```

Appendix Figure S4. The enolase sequences of Homo sapiens, *Acinetobacter baumannii* and *Galleria mellonella*.

Appendix Table S1

Appendix Table S1. Plasmids and primers used in this study.

Plasmids, and primers	Relevant features and use	References
**pGEM-T**	Suicide plasmid for *A. baumannii*. Amp^R	Promega (Spain)
**enolase intUp**	5-TGACGGCGACAAATCTCGTT-3	Present study
**enolase intLw**	5-GCGCAAAACCACCTTCATCA-3	Present study
**enolase extUp**	5-CTTGAGCCATTCTTGGCACA-3	Present study
**enolase extLw**	5-TTAAAAACCCGGCGCTTAGG-3	Present study
**M13F**	5-GTAAAACGACGGCCAGT-3	Smani et al. 2013
**M13R**	5-CAGGAAACAGCTATGAC-3	Smani et al. 2013

Appendix Table S2

Appendix Table S2. EIIP and AQVN parameters to analyze the electronic properties of drugs tested for synergy against *A. baumannii* Ab ATCC 17978 strain

Molecule	AQVN	EIIP
ENOblock	2.628	0.078
Colistin	2.592	0.084
Imipenem	2.973	0.034
Ceftazidime	3.288	0.127
Tigecycline	2.815	0.025

AQVN: and average quasi-valence number, EIIP: Electron-ion interaction potential.

Appendix Table S3

Appendix Table S3. Antibacterial activity of ENOblock in colistin-resistant *E. coli* strains.

Strain#	Colistin resistance mechanism	Colistin MIC (mg/L)	ENOblock MIC (mg/L)
**CRA5**	-	8	16
**CRA7**	-	8	16
**CRA8**	MCR1	8	16
**CRA17**	-	8	32
**CRA20**	MCR1	8	32
**CRA32**	-	8	8
**CRA57**	-	4	16
**R2**	MCR1	8	32
**R5**	MCR1	4	32
**R6**	MCR1	8	16
**R34**	MCR1.5	8	64
**R37**	MCR3.2	8	32
**MCR1+**	MCR1	4	64

Appendix Table S4

Appendix Table S4. Antibacterial activity of ENOblock in carbapenem-resistant *K. pneumonaie* strains.

**Strain#**	**Meropenem resistance mechanism**	**Meropenem MIC (mg/L)**	**ENOblock MIC (mg/L)**
**Kp6**	KPC-3	2	32
**Kp7**	KPC-2	-	16
**Kp8**	KPC-2	-	64
**Kp9**	KPC-41	-	64
**Kp10**	KPC-2	16	32
**Kp11**	KPC-2	4	16
**Kp12**	KPC-2	16	64
**Kp13**	KPC-3	>16	64
**Kp14**	KPC-2	4	64
**Kp15**	KPC-11	4	64
**Kp16**	KPC-3	>16	64
**Kp17**	KPC-3	16	16
**Kp18**	KPC-2	4	32
**Kp19**	KPC-3	4	64

Appendix Table S5

Appendix Table S5. ISM frequencies for ENOblock intermolecular interactions and corresponding signal – to – noise (S/N) ratios.

Interaction	F	S/N
Enolase - ENOblock	0.271	12.44
	0.435	3.40
Fibronectin - enolase - ENOblock	0.271	51.498
	0.435	11.96
Fibrinogen - enolase - ENOblock	0.271	18.161
	0.435	8.78
