# Supplementary material for: ENOblock synergizes with colistin to treat Acinetobacter baumannii infections
Source: EMBO Mol Med. 2025 Oct 31;17(12):3496–524. doi: 10.1038/s44321-025-00331-2 (PMC12686454; doi:10.1038/s44321-025-00331-2)
Supplement: Supplementary file 6 — Source data Fig. 2 [file 44321_2025_331_MOESM6_ESM.zip › FIGURE 2/2B/EIIP and AQVN parameters .docx]

**Table S2.** EIIP and AQVN parameters to analyze the electronic properties of drugs tested for synergy against *A. baumannii* Ab ATCC 17978 strain

| **Molecule** | **AQVN** | **EIIP** |
| --- | --- | --- |
| ENOblock | 2.628 | 0.078 |
| Colistin | 2.592 | 0.084 |
| Imipenem | 2.973 | 0.034 |
| Ceftazidime | 3.288 | 0.127 |
| Tigecycline | 2.815 | 0.025 |

AQVN: and average quasi-valence number, EIIP: Electron-ion interaction potential.
