# Supplementary figures and images for: ENOblock synergizes with colistin to treat Acinetobacter baumannii infections
Source: EMBO Mol Med. 2025 Oct 31;17(12):3496–524. doi: 10.1038/s44321-025-00331-2 (PMC12686454; doi:10.1038/s44321-025-00331-2)

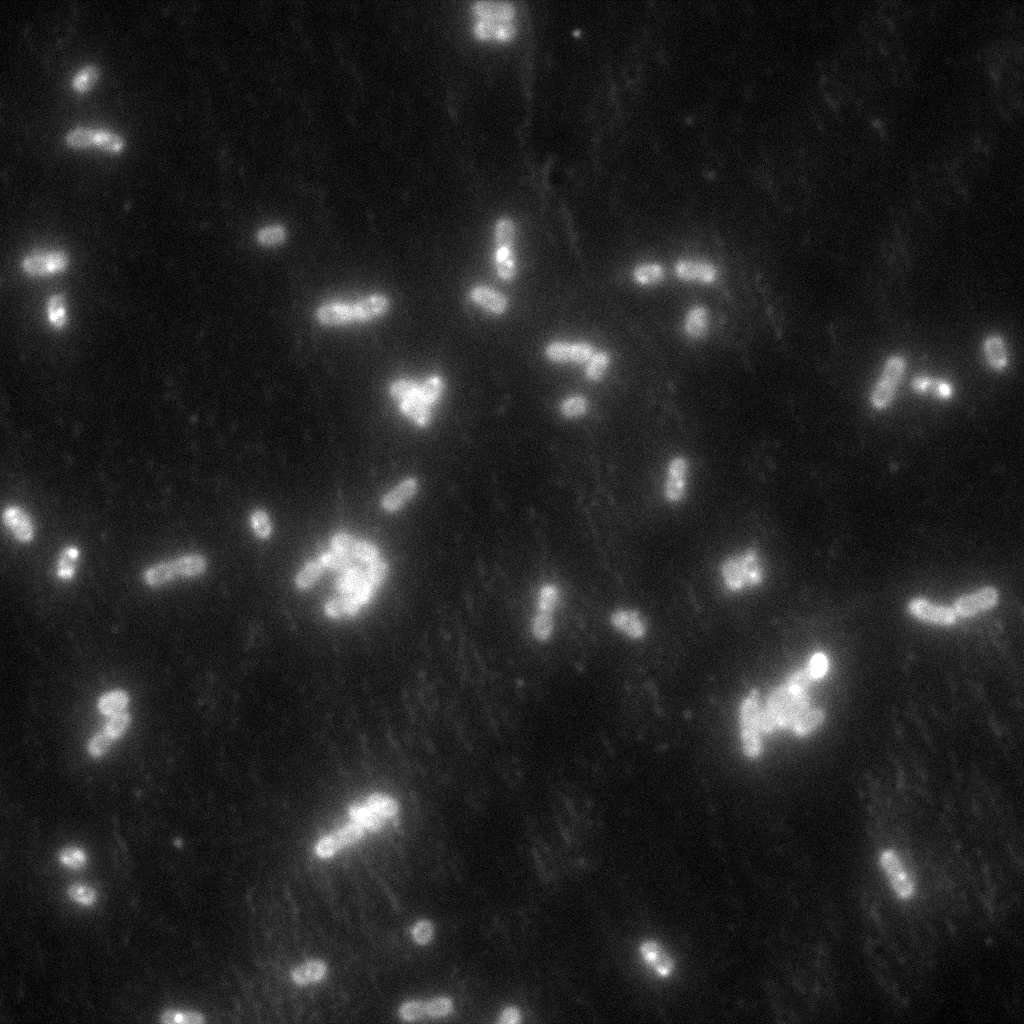

Supplement: Supplementary file 7 — Source data Fig. 3 [file 44321_2025_331_MOESM7_ESM.zip › FIGURE 3/3A/ENOblock.tif]

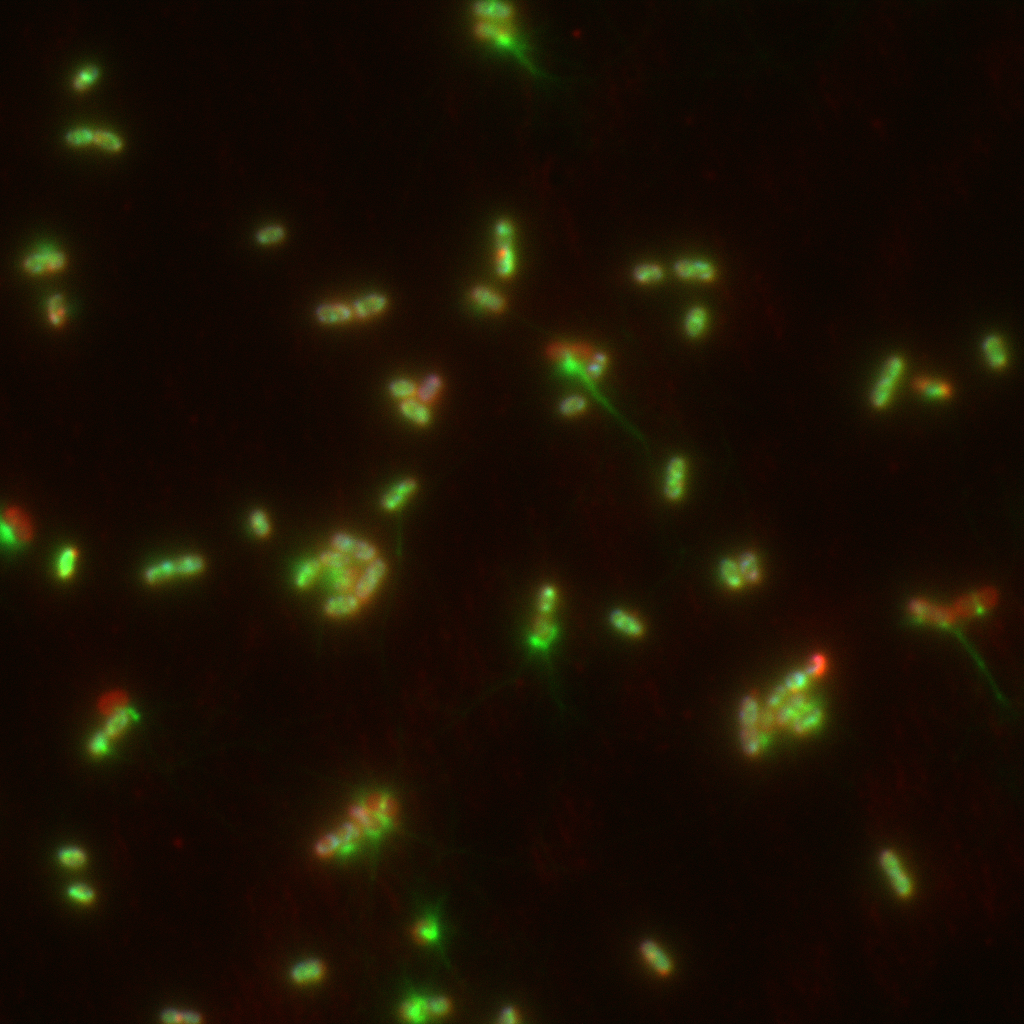

Supplement: Supplementary file 7 — Source data Fig. 3 [file 44321_2025_331_MOESM7_ESM.zip › FIGURE 3/3A/ENOblock.tif (RGB).tif]

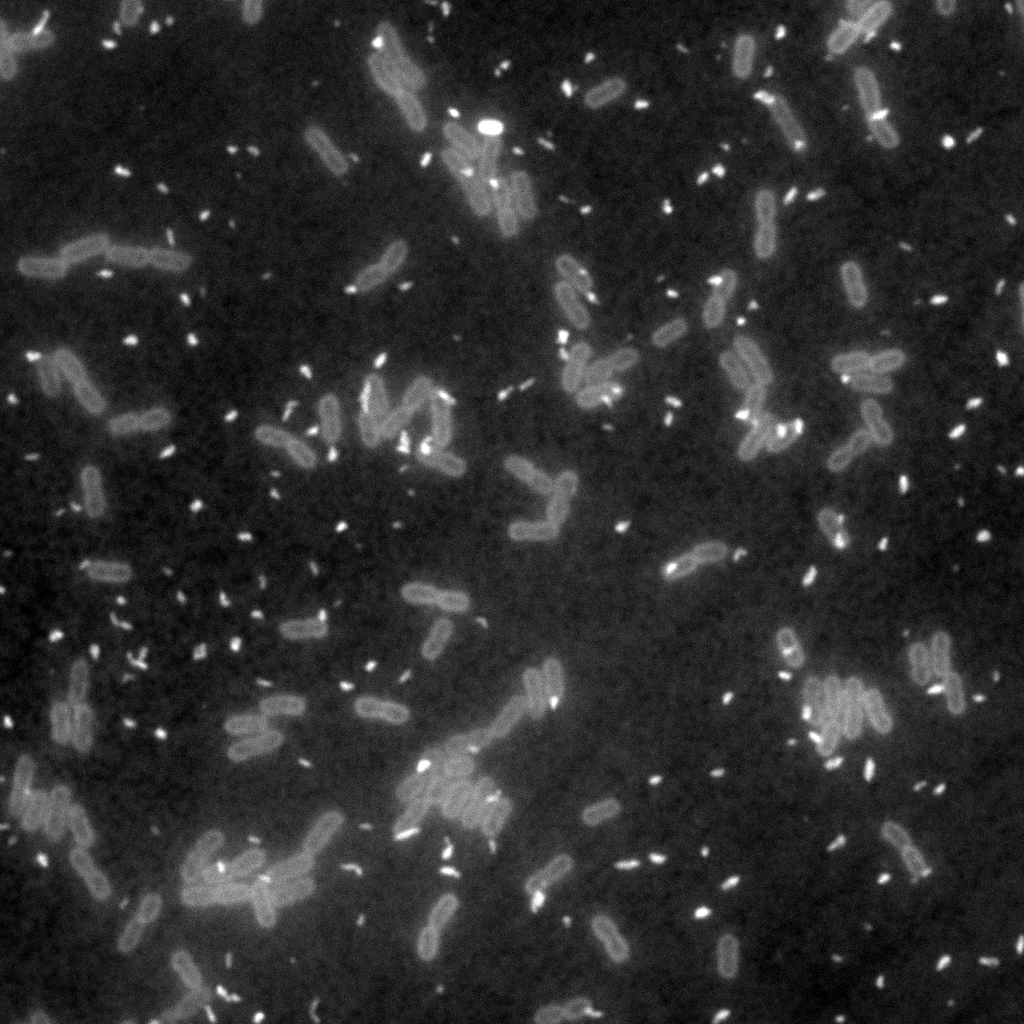

Supplement: Supplementary file 7 — Source data Fig. 3 [file 44321_2025_331_MOESM7_ESM.zip › FIGURE 3/3A/Untreated.tif]

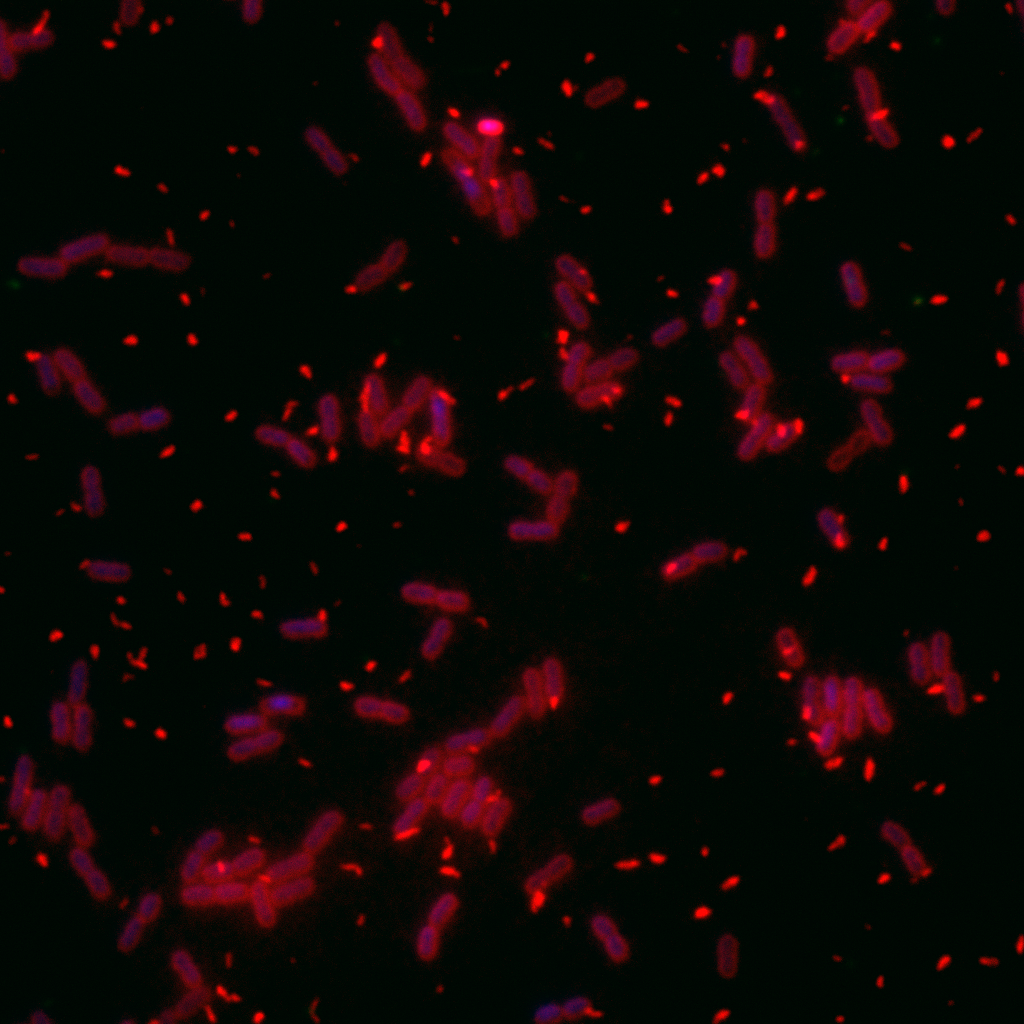

Supplement: Supplementary file 7 — Source data Fig. 3 [file 44321_2025_331_MOESM7_ESM.zip › FIGURE 3/3A/Untreated.tif (RGB).tif]

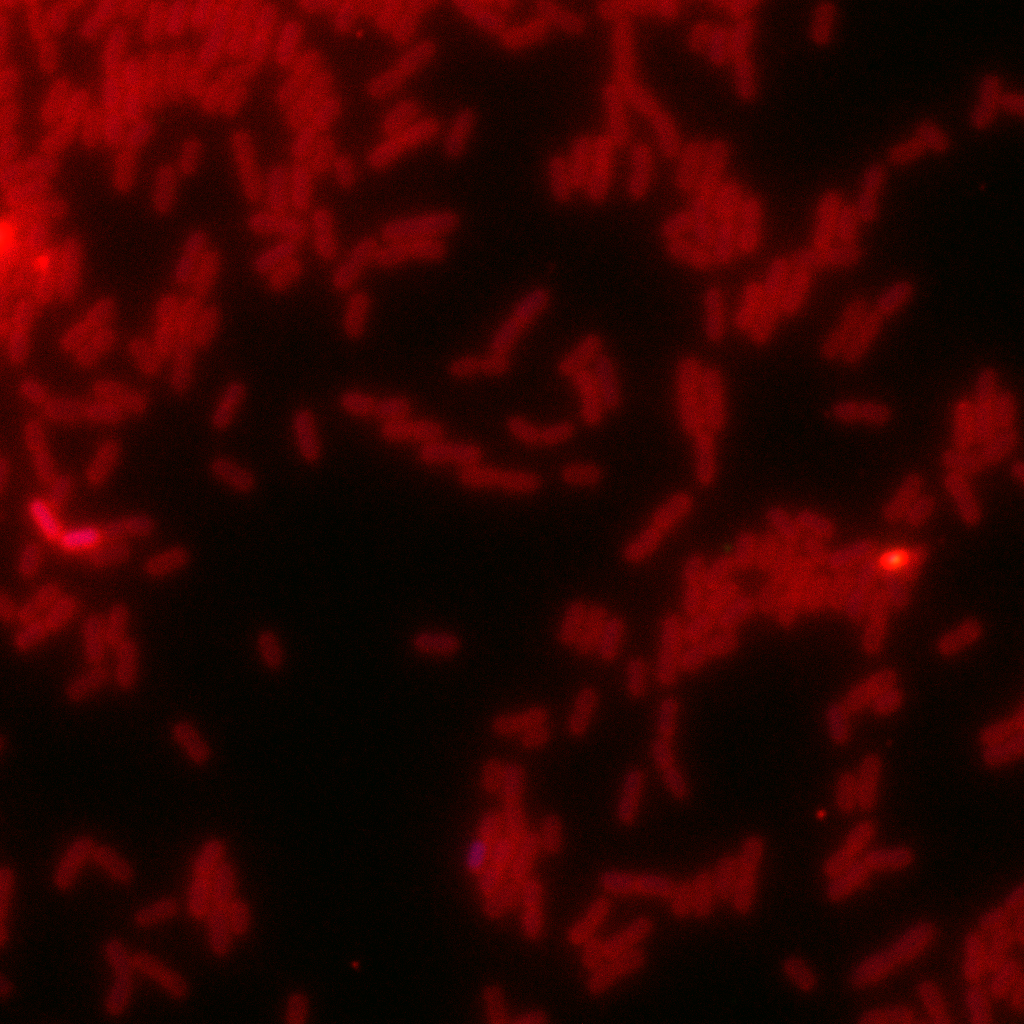

Supplement: Supplementary file 7 — Source data Fig. 3 [file 44321_2025_331_MOESM7_ESM.zip › FIGURE 3/3C/RGB/60 min Untreated.tif (RGB).tif]

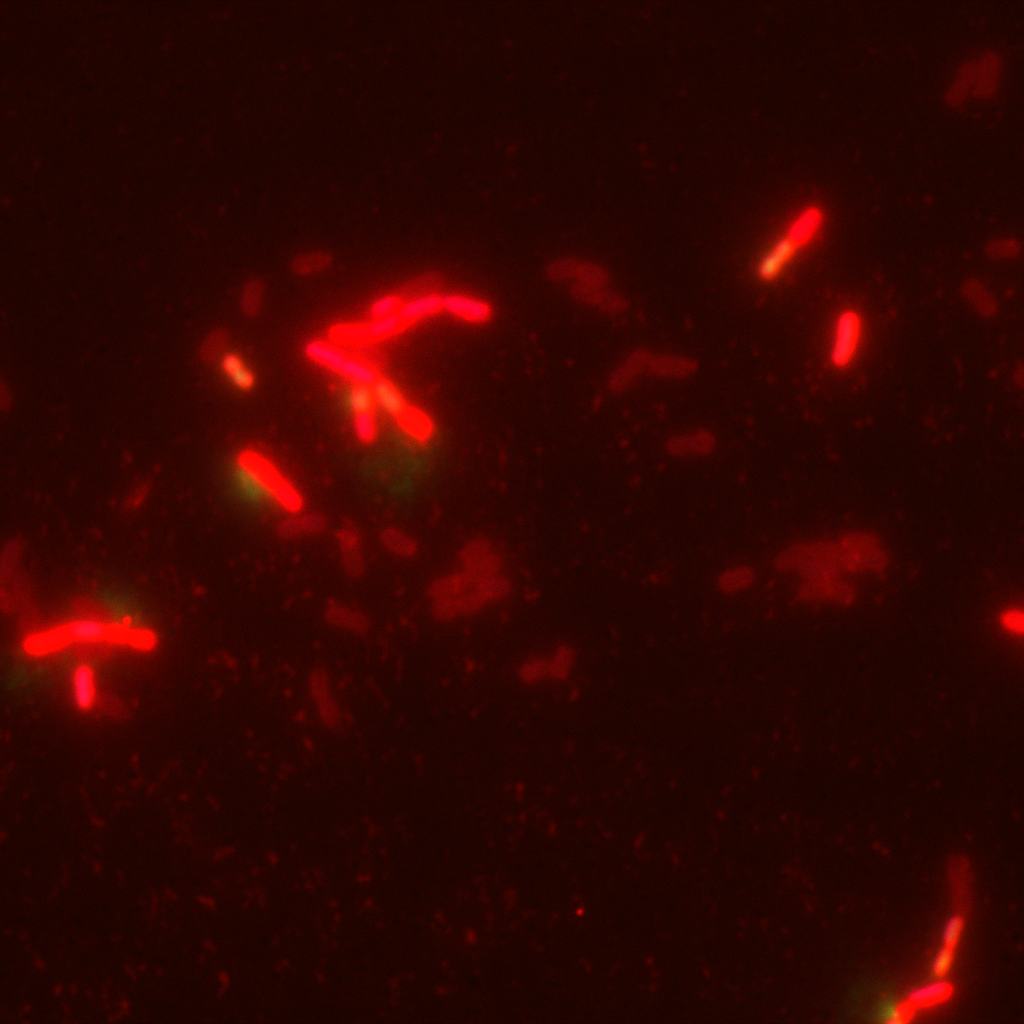

Supplement: Supplementary file 7 — Source data Fig. 3 [file 44321_2025_331_MOESM7_ESM.zip › FIGURE 3/3C/RGB/10 min ENOblock 8 ug.tif (RGB).tif]

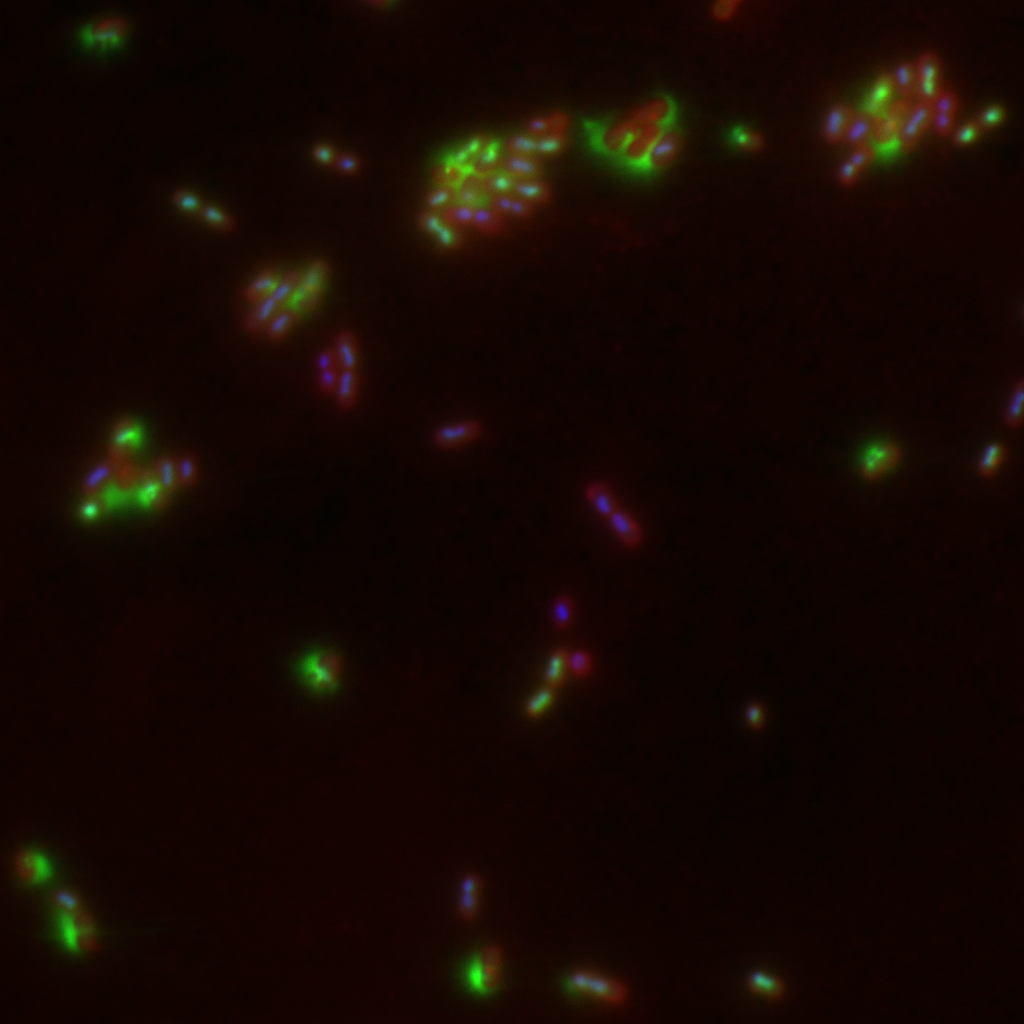

Supplement: Supplementary file 7 — Source data Fig. 3 [file 44321_2025_331_MOESM7_ESM.zip › FIGURE 3/3C/RGB/10 min Colistin .tif (RGB).tif]

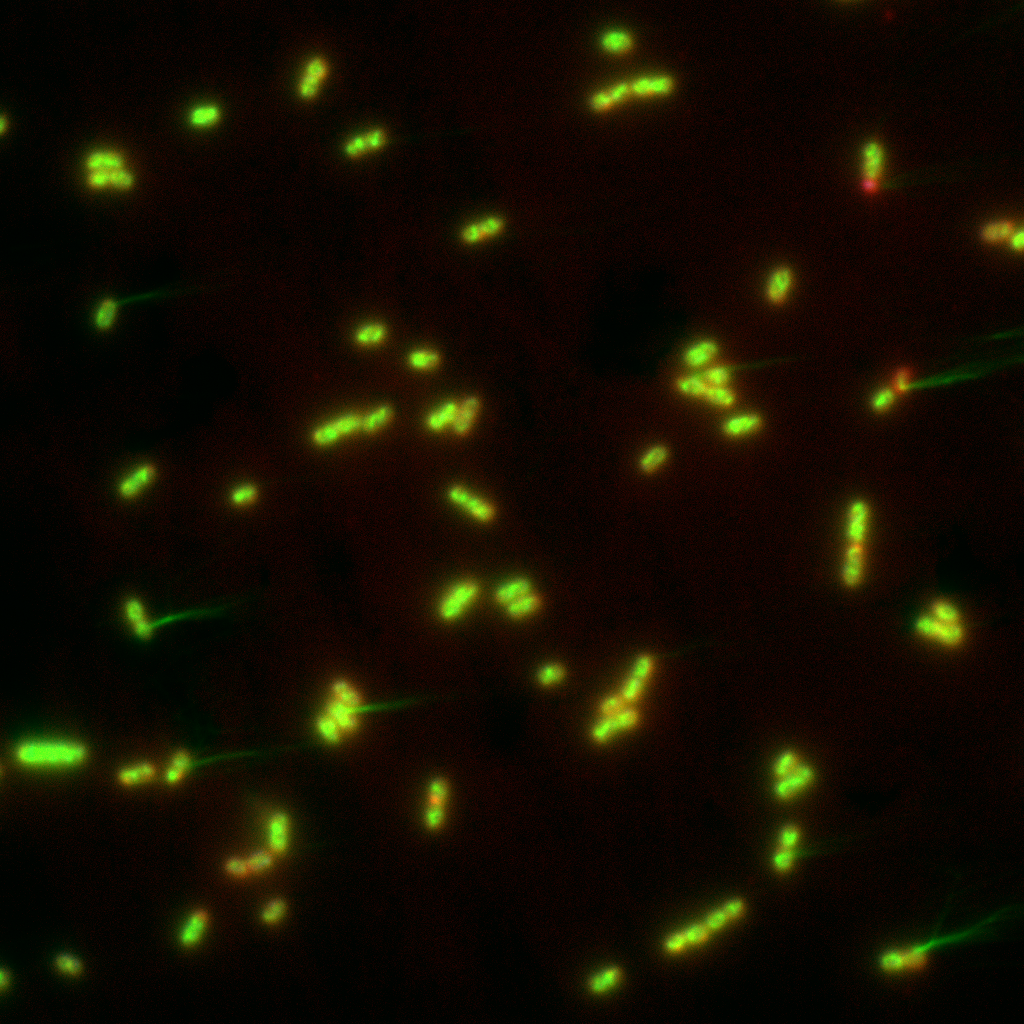

Supplement: Supplementary file 7 — Source data Fig. 3 [file 44321_2025_331_MOESM7_ESM.zip › FIGURE 3/3C/RGB/60 min ENOblock 32 ug.tif (RGB).tif]

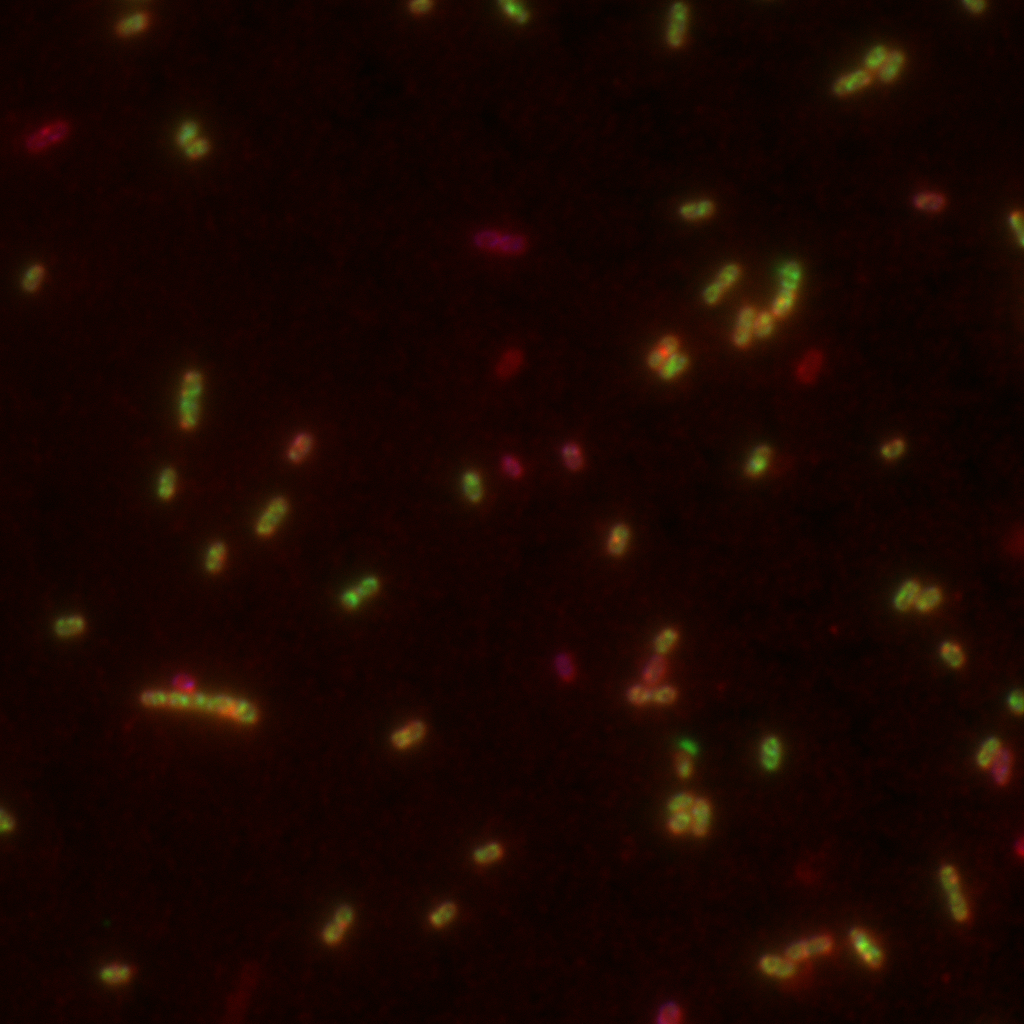

Supplement: Supplementary file 7 — Source data Fig. 3 [file 44321_2025_331_MOESM7_ESM.zip › FIGURE 3/3C/RGB/60 min ENOblock 16 ug.tif (RGB).tif]

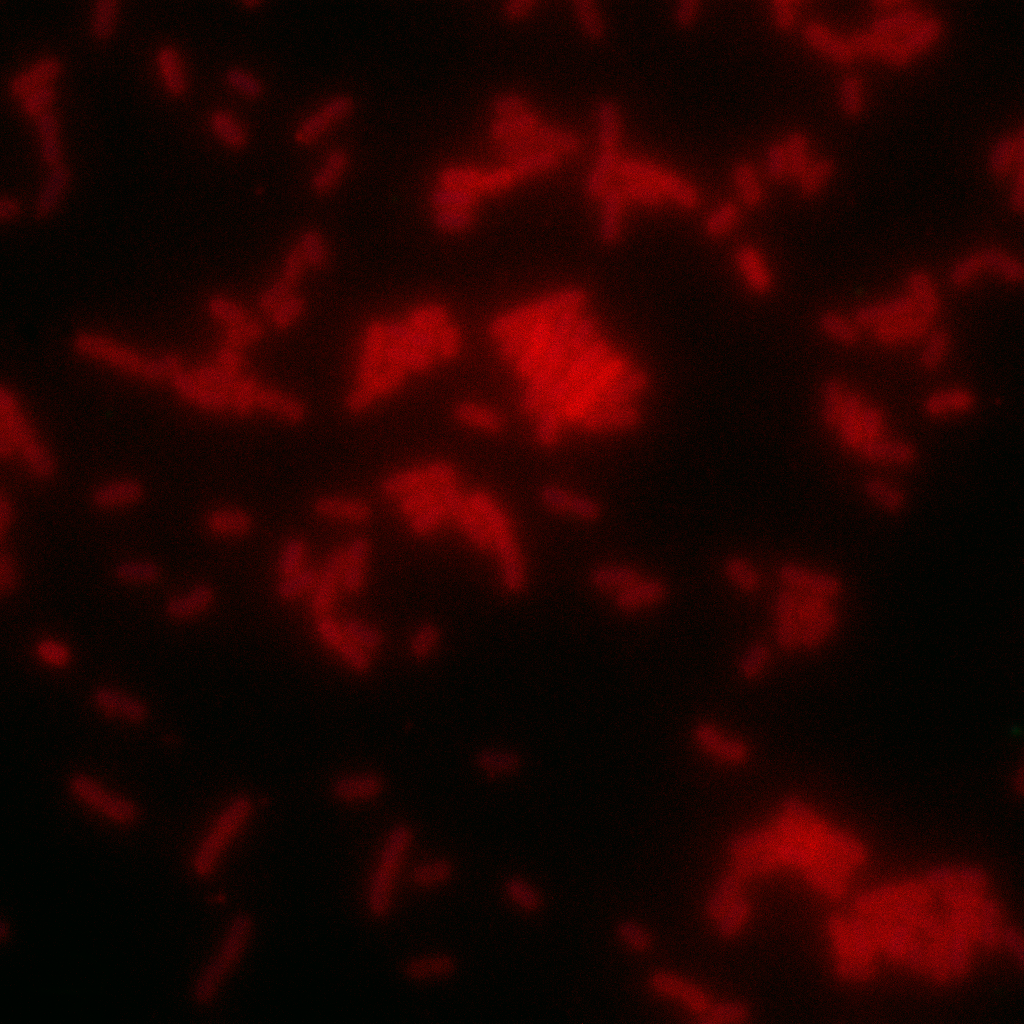

Supplement: Supplementary file 7 — Source data Fig. 3 [file 44321_2025_331_MOESM7_ESM.zip › FIGURE 3/3C/RGB/30 min Untreated.tif (RGB).tif]

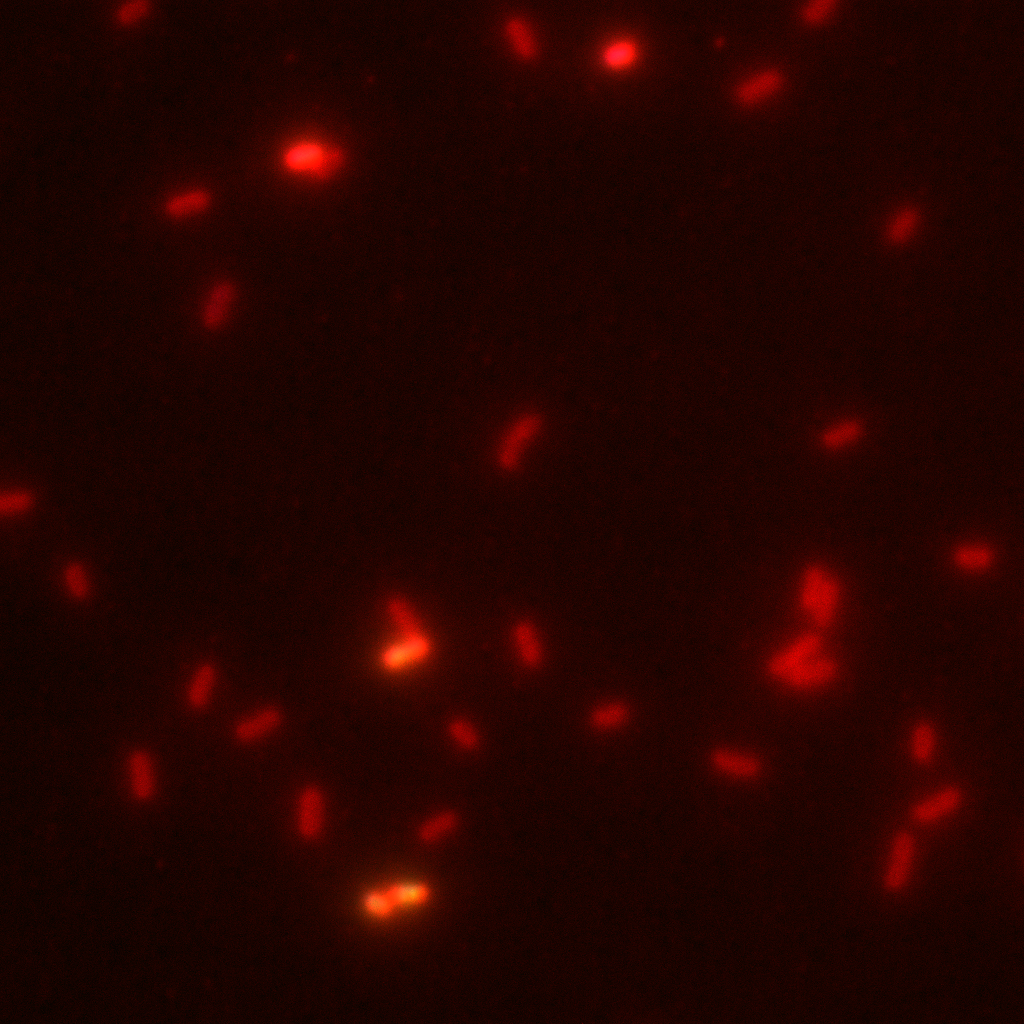

Supplement: Supplementary file 7 — Source data Fig. 3 [file 44321_2025_331_MOESM7_ESM.zip › FIGURE 3/3C/RGB/30 min ENOblock 8 ug.tif (RGB).tif]

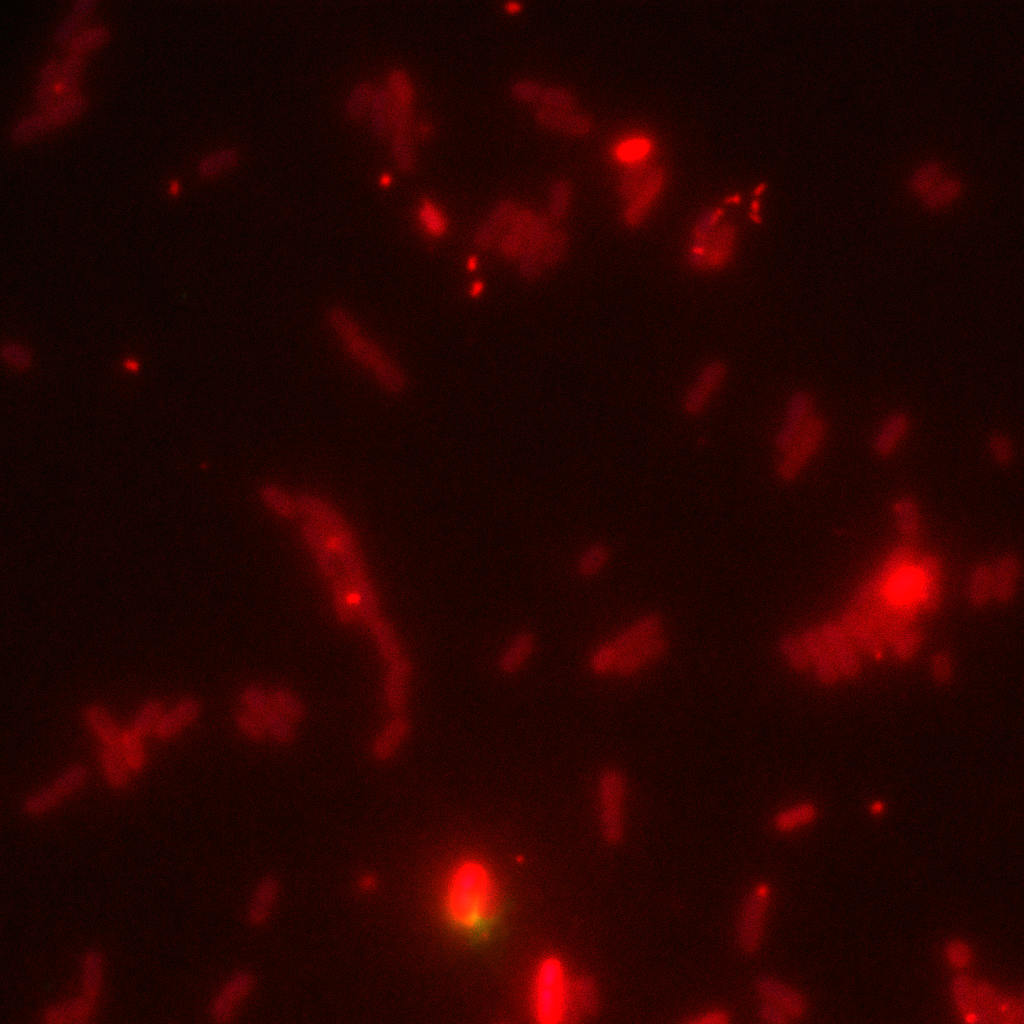

Supplement: Supplementary file 7 — Source data Fig. 3 [file 44321_2025_331_MOESM7_ESM.zip › FIGURE 3/3C/RGB/10 min Untreated.tif (RGB).tif]

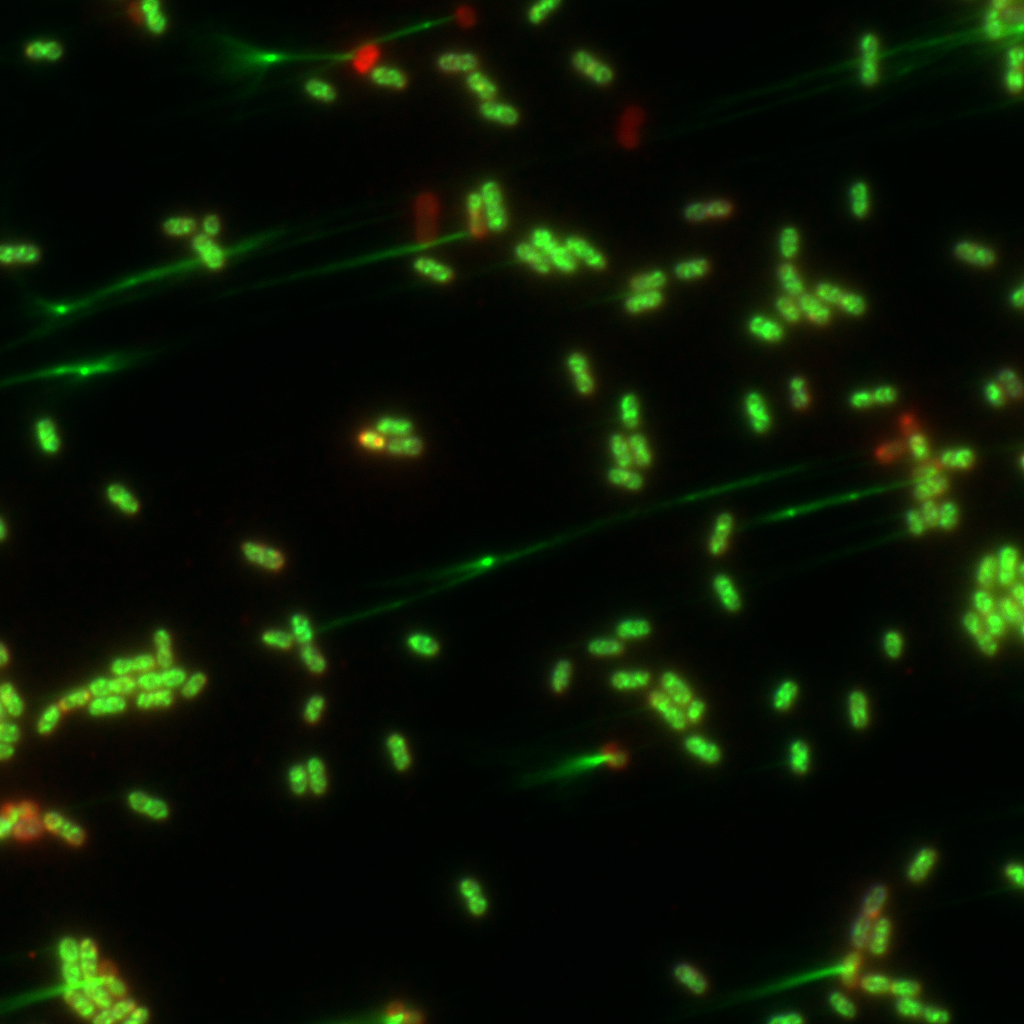

Supplement: Supplementary file 7 — Source data Fig. 3 [file 44321_2025_331_MOESM7_ESM.zip › FIGURE 3/3C/RGB/10 min Enoblock 32 ug.tif (RGB).tif]

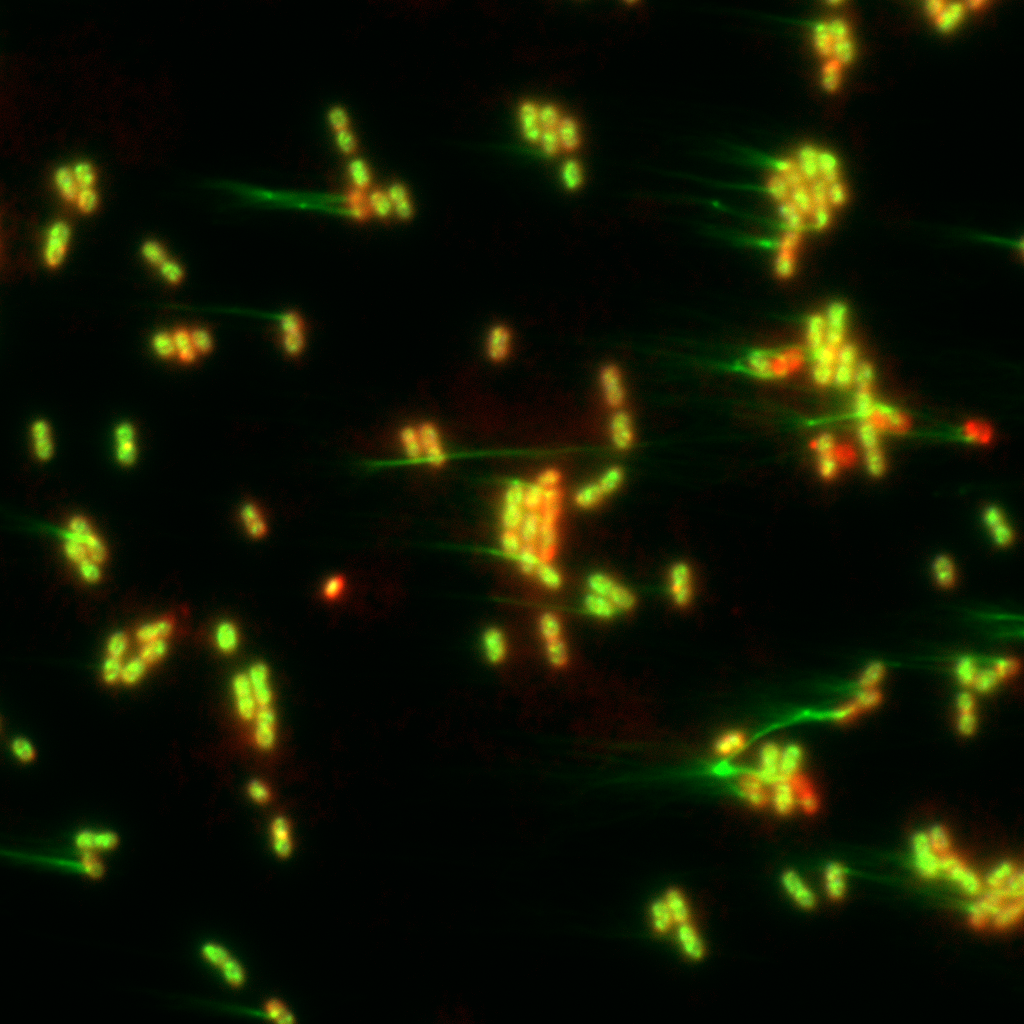

Supplement: Supplementary file 7 — Source data Fig. 3 [file 44321_2025_331_MOESM7_ESM.zip › FIGURE 3/3C/RGB/30 min ENOblock 32 ug.tif (RGB).tif]

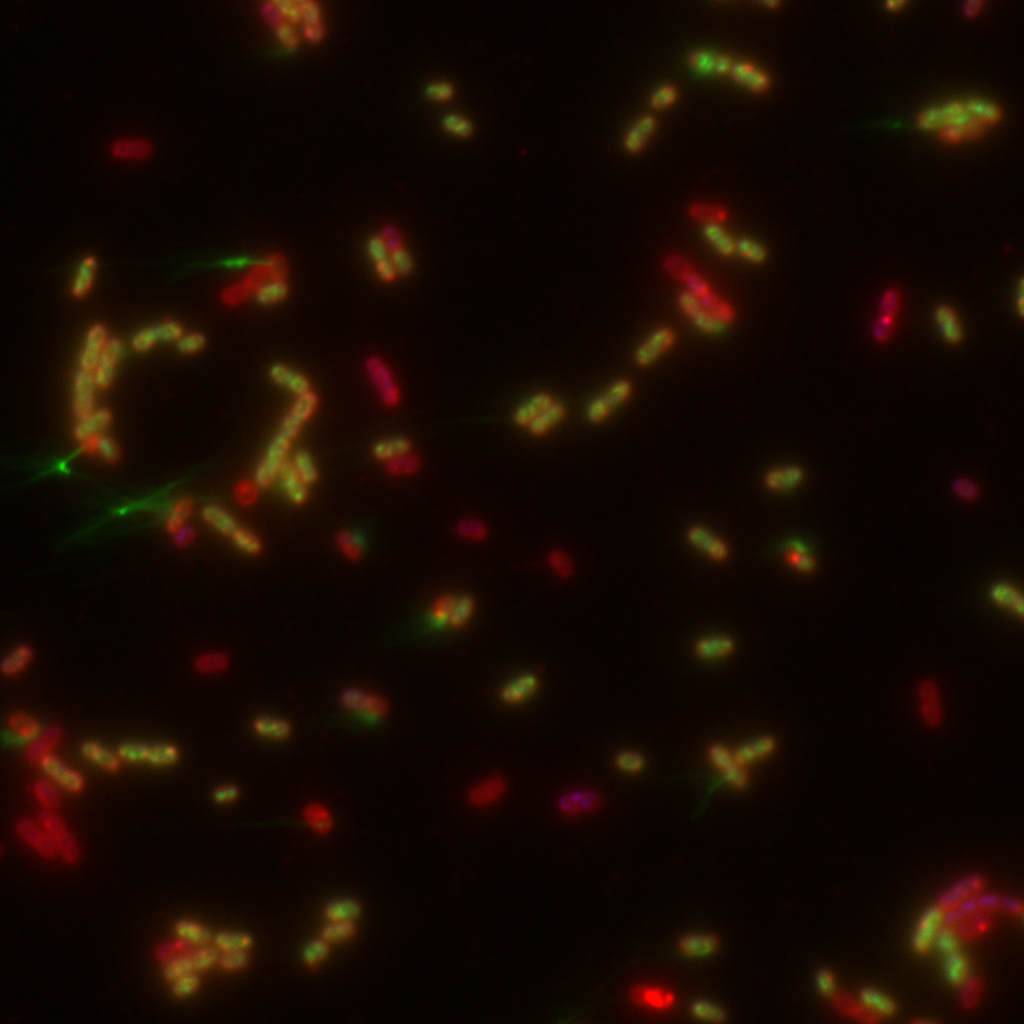

Supplement: Supplementary file 7 — Source data Fig. 3 [file 44321_2025_331_MOESM7_ESM.zip › FIGURE 3/3C/RGB/30 min ENOblock 16 ug.tif (RGB).tif]

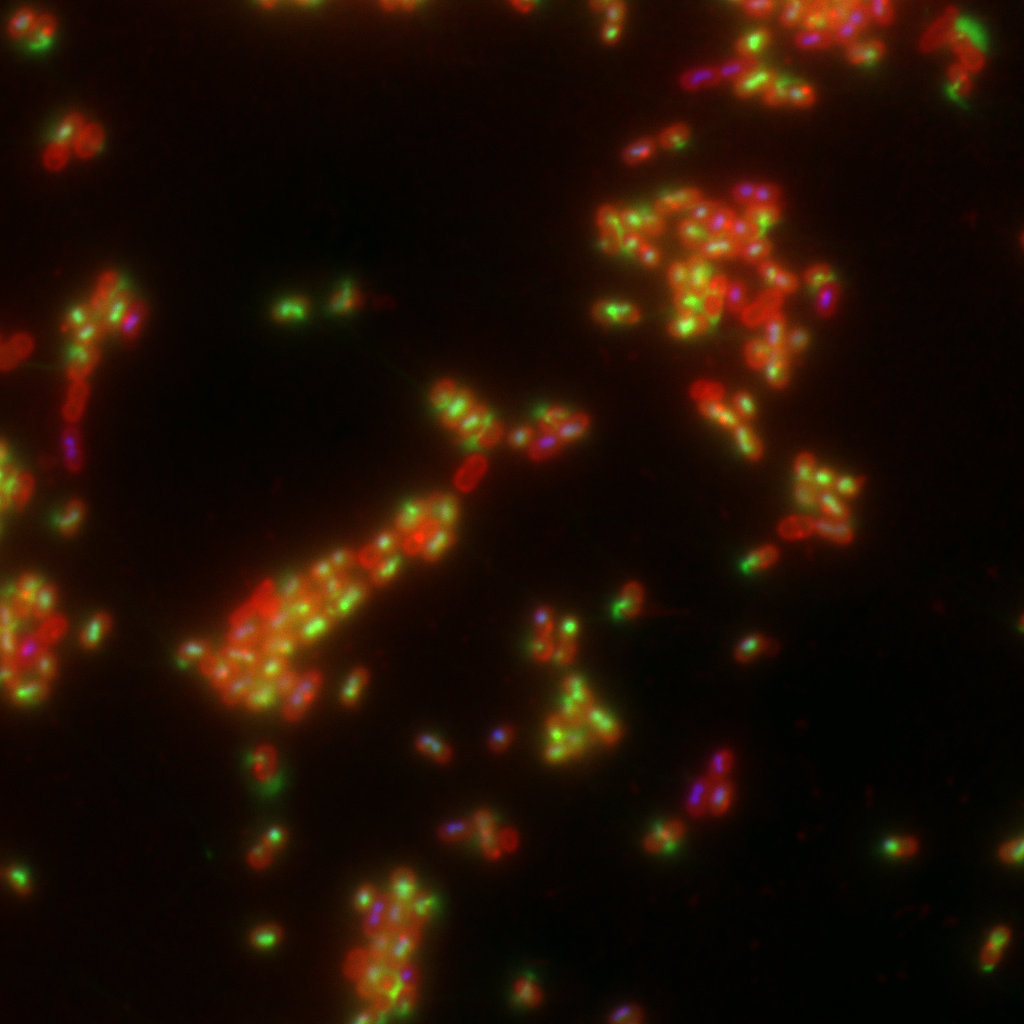

Supplement: Supplementary file 7 — Source data Fig. 3 [file 44321_2025_331_MOESM7_ESM.zip › FIGURE 3/3C/RGB/60 min Colistin.tif (RGB).tif]

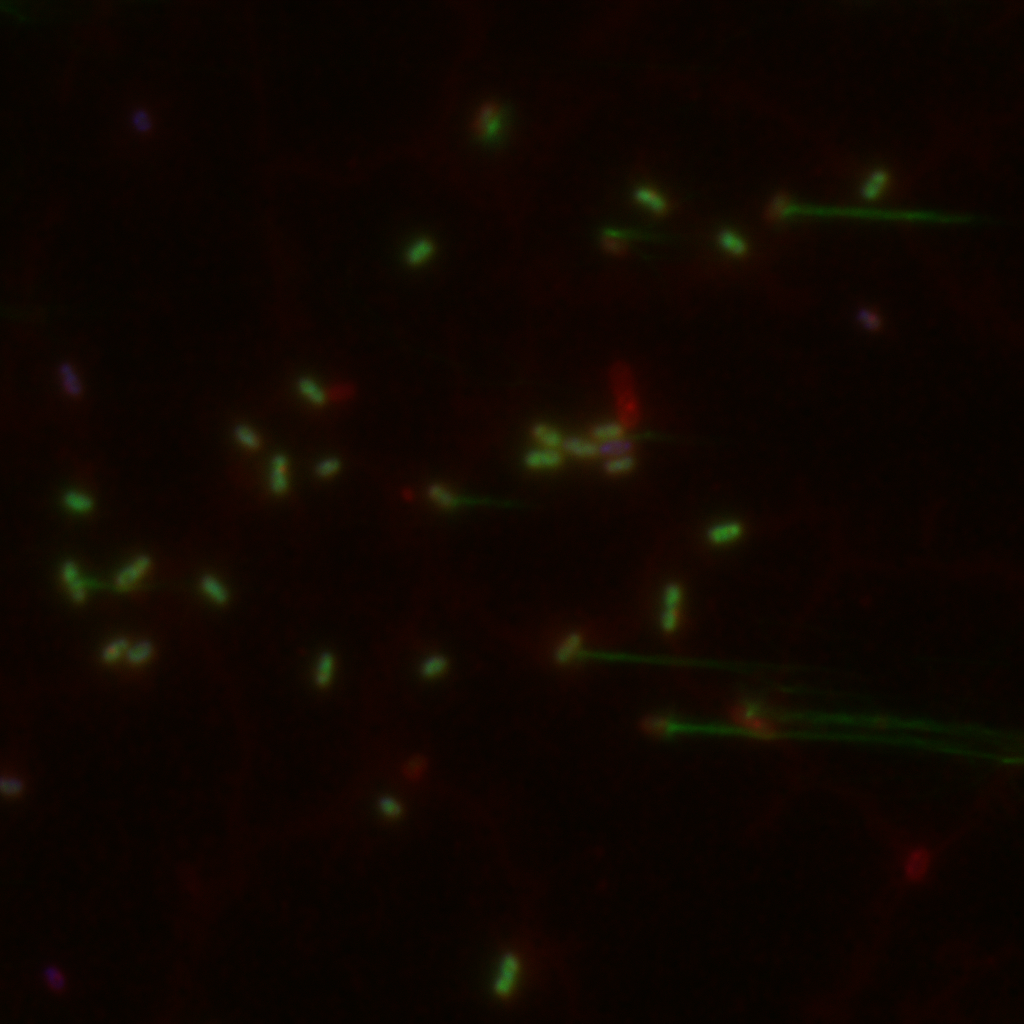

Supplement: Supplementary file 7 — Source data Fig. 3 [file 44321_2025_331_MOESM7_ESM.zip › FIGURE 3/3C/RGB/10 min ENOblock 16 ug.tif (RGB).tif]

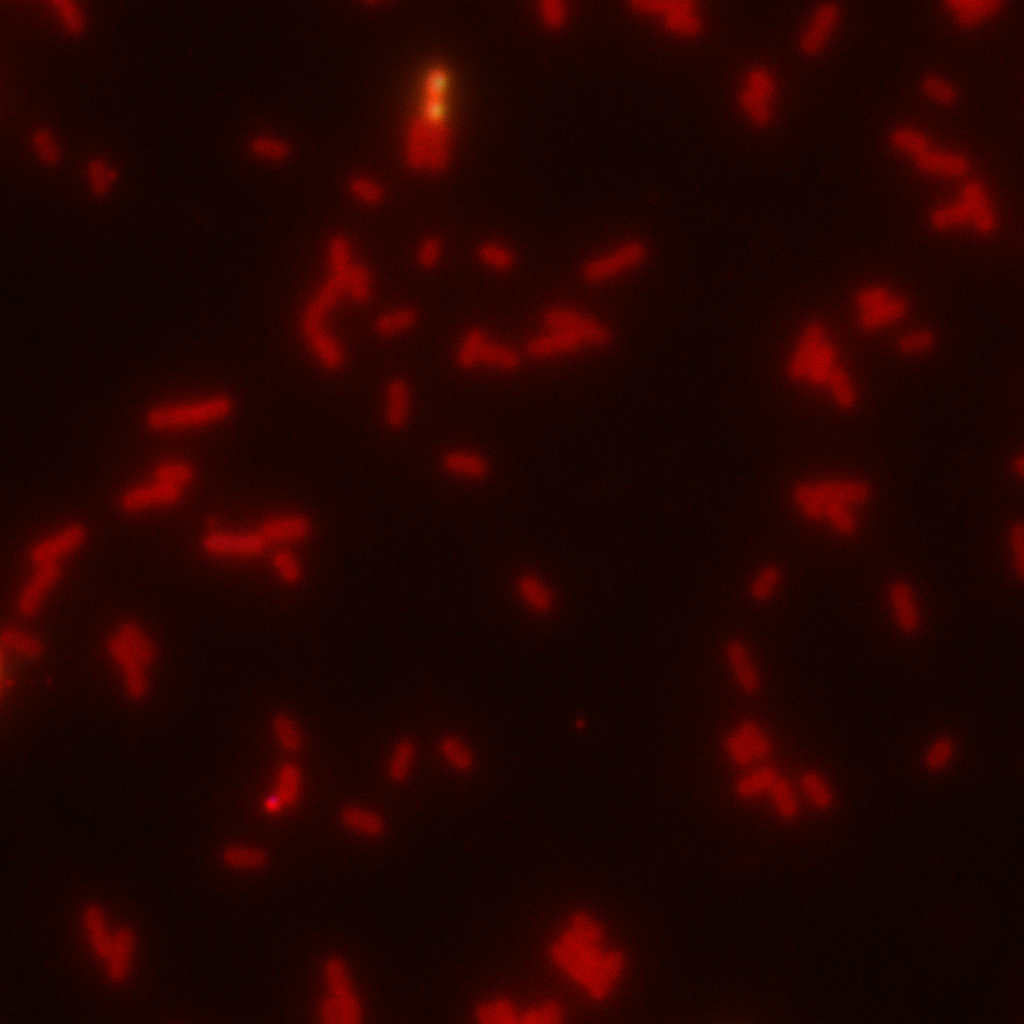

Supplement: Supplementary file 7 — Source data Fig. 3 [file 44321_2025_331_MOESM7_ESM.zip › FIGURE 3/3C/RGB/60 min ENOblock 8 ug.tif (RGB).tif]

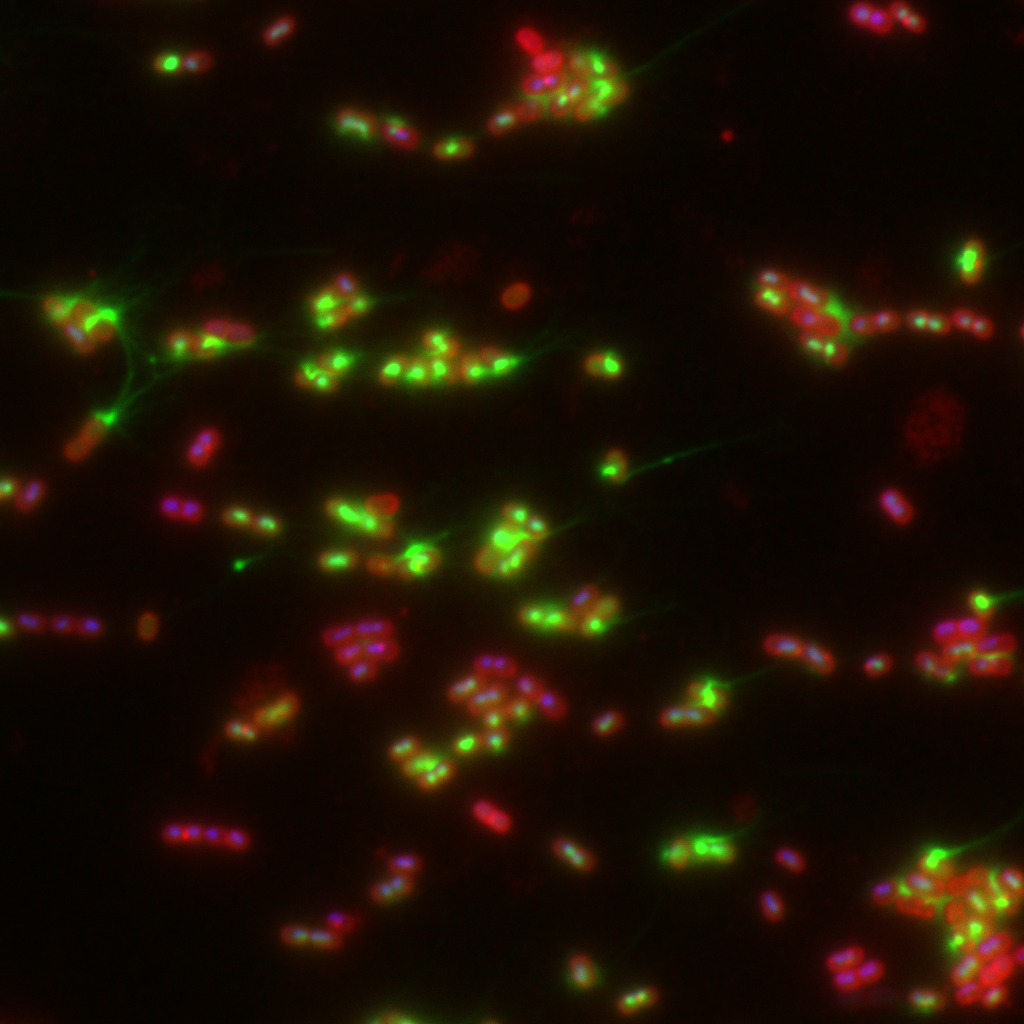

Supplement: Supplementary file 7 — Source data Fig. 3 [file 44321_2025_331_MOESM7_ESM.zip › FIGURE 3/3C/RGB/30 min Colistin.tif (RGB).tif]

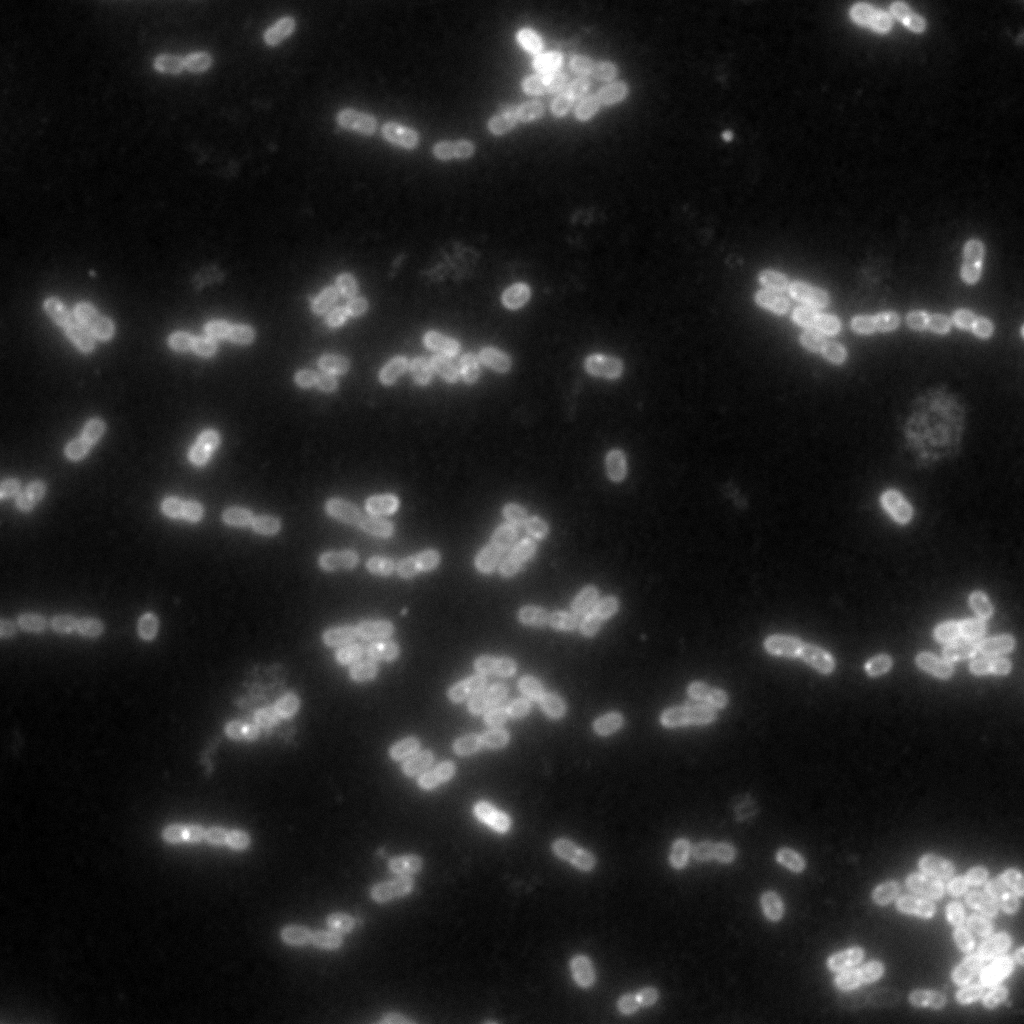

Supplement: Supplementary file 7 — Source data Fig. 3 [file 44321_2025_331_MOESM7_ESM.zip › FIGURE 3/3C/TIFF/30 min Colistin.tif]

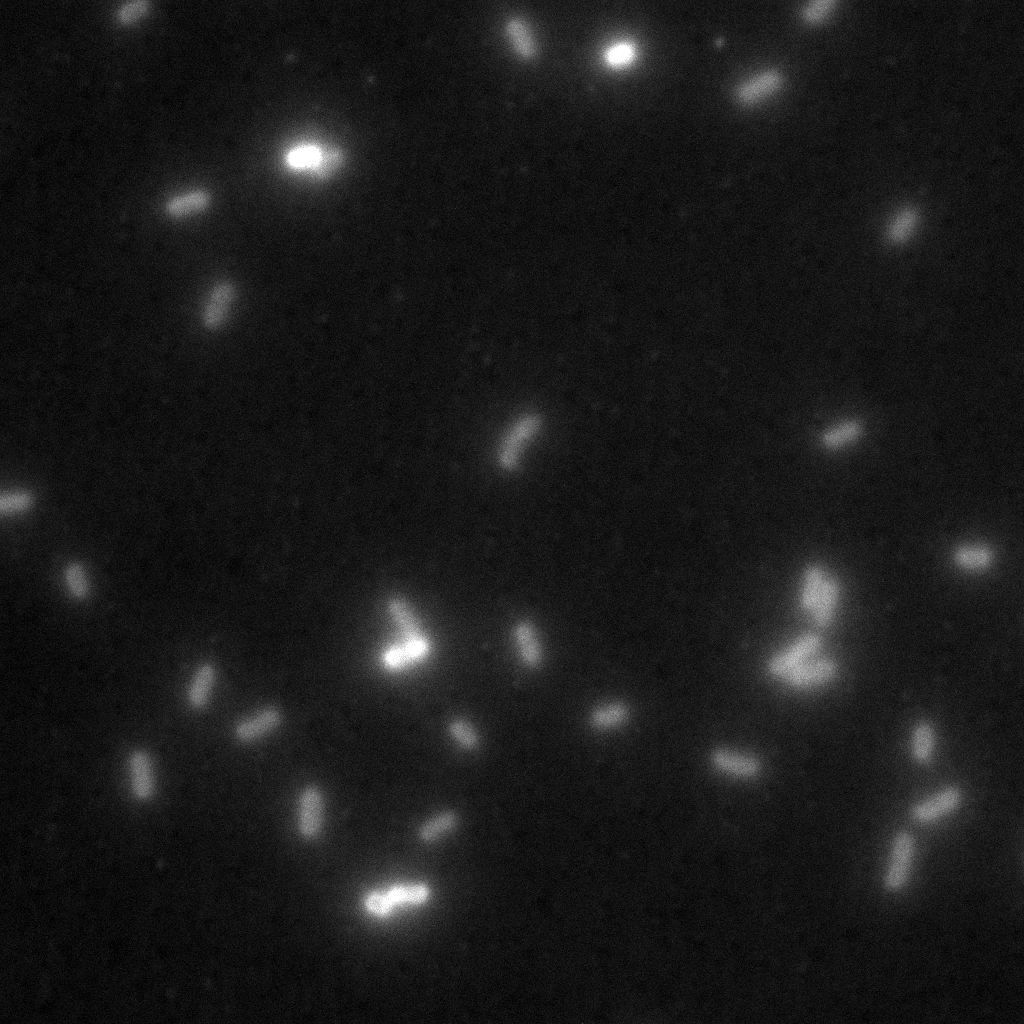

Supplement: Supplementary file 7 — Source data Fig. 3 [file 44321_2025_331_MOESM7_ESM.zip › FIGURE 3/3C/TIFF/30 min ENOblock 8 ug.tif]

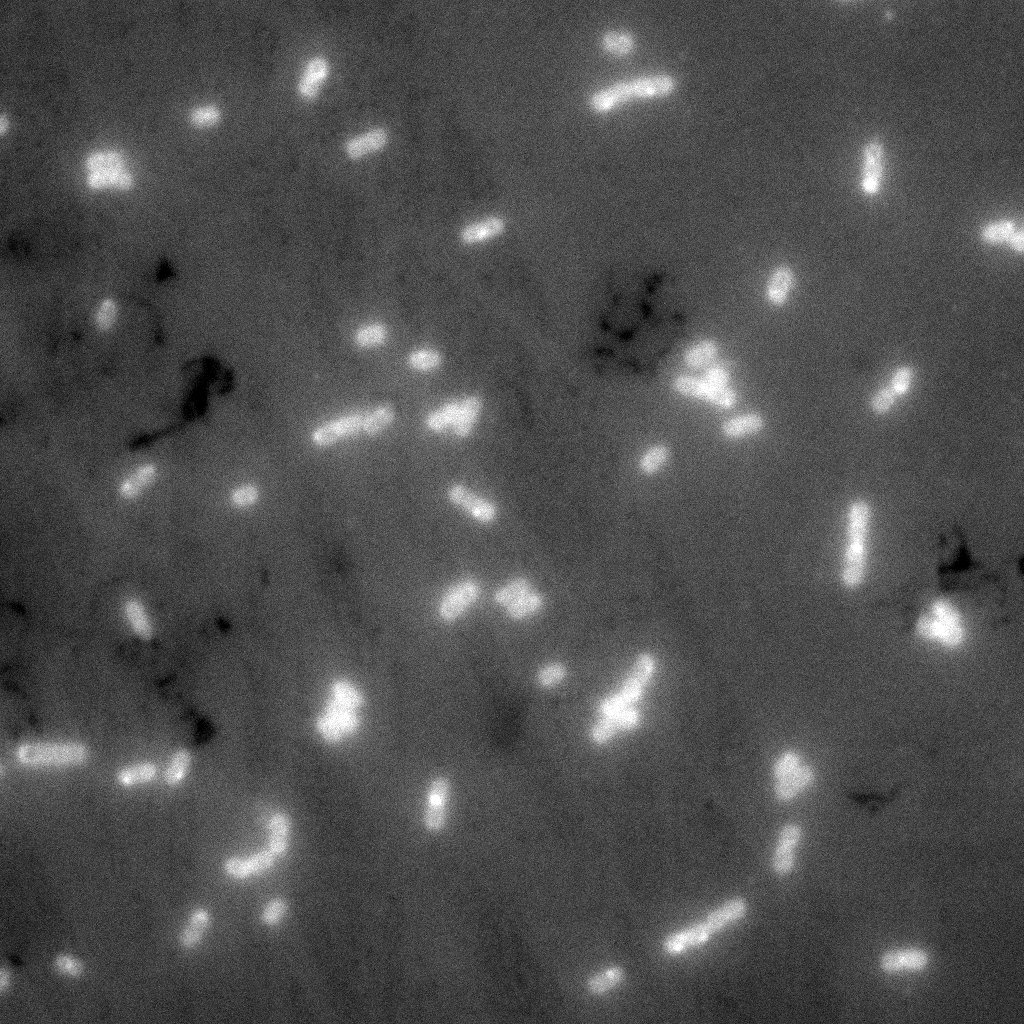

Supplement: Supplementary file 7 — Source data Fig. 3 [file 44321_2025_331_MOESM7_ESM.zip › FIGURE 3/3C/TIFF/60 min ENOblock 32 ug.tif]

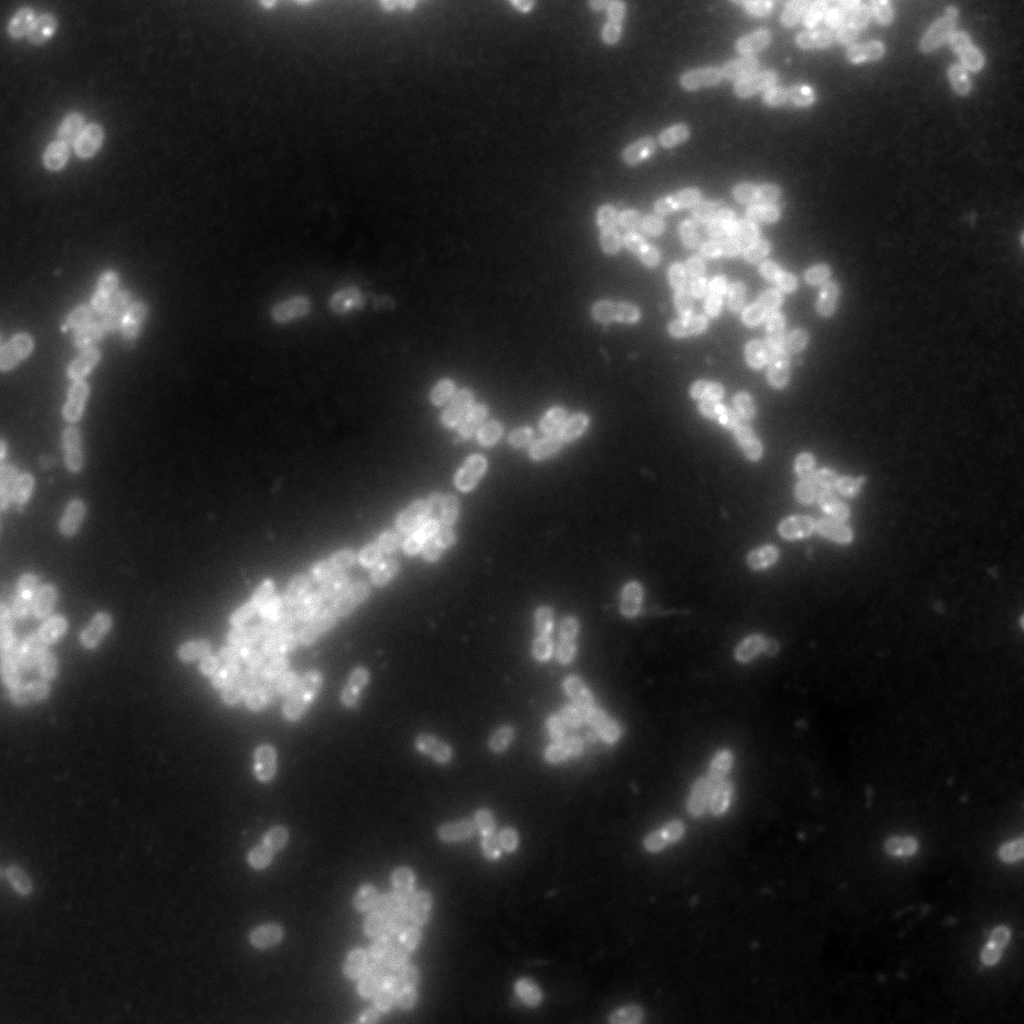

Supplement: Supplementary file 7 — Source data Fig. 3 [file 44321_2025_331_MOESM7_ESM.zip › FIGURE 3/3C/TIFF/60 min Colistin.tif]

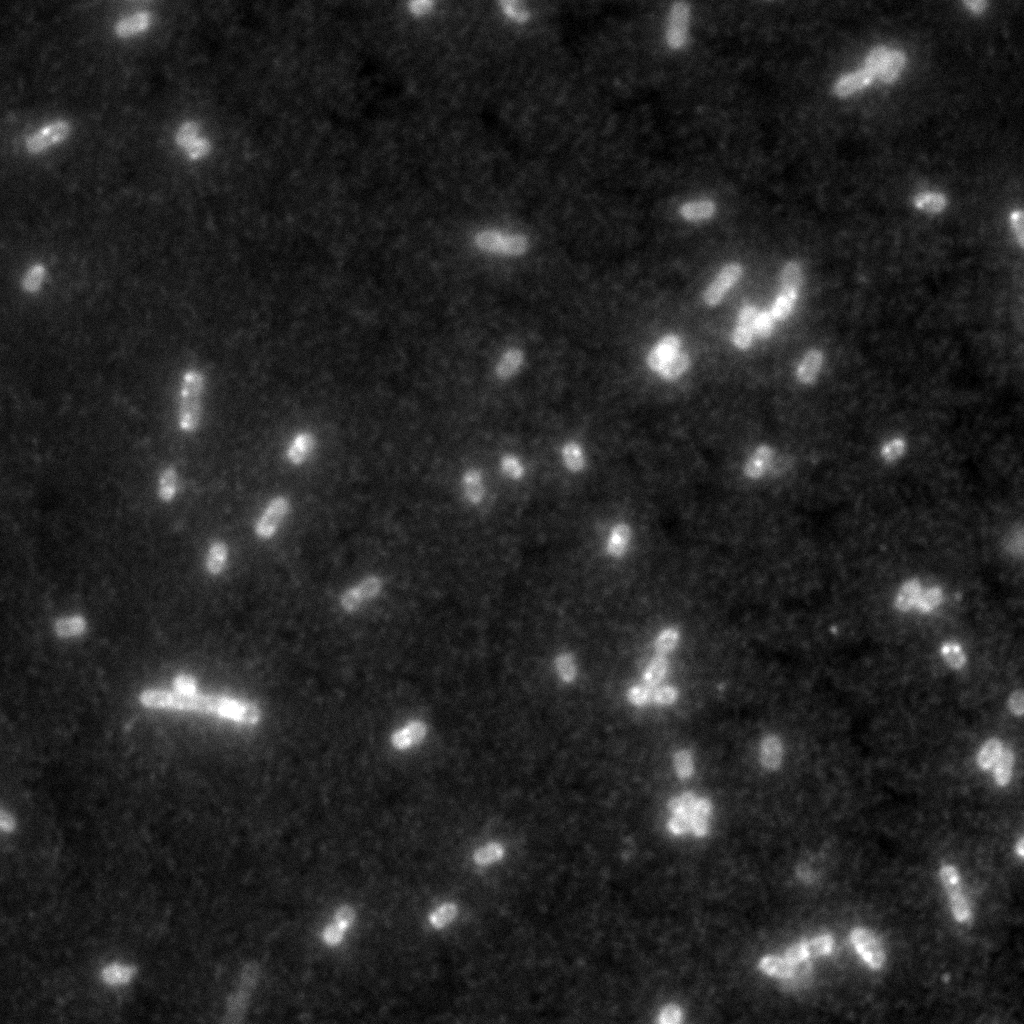

Supplement: Supplementary file 7 — Source data Fig. 3 [file 44321_2025_331_MOESM7_ESM.zip › FIGURE 3/3C/TIFF/60 min ENOblock 16 ug.tif]

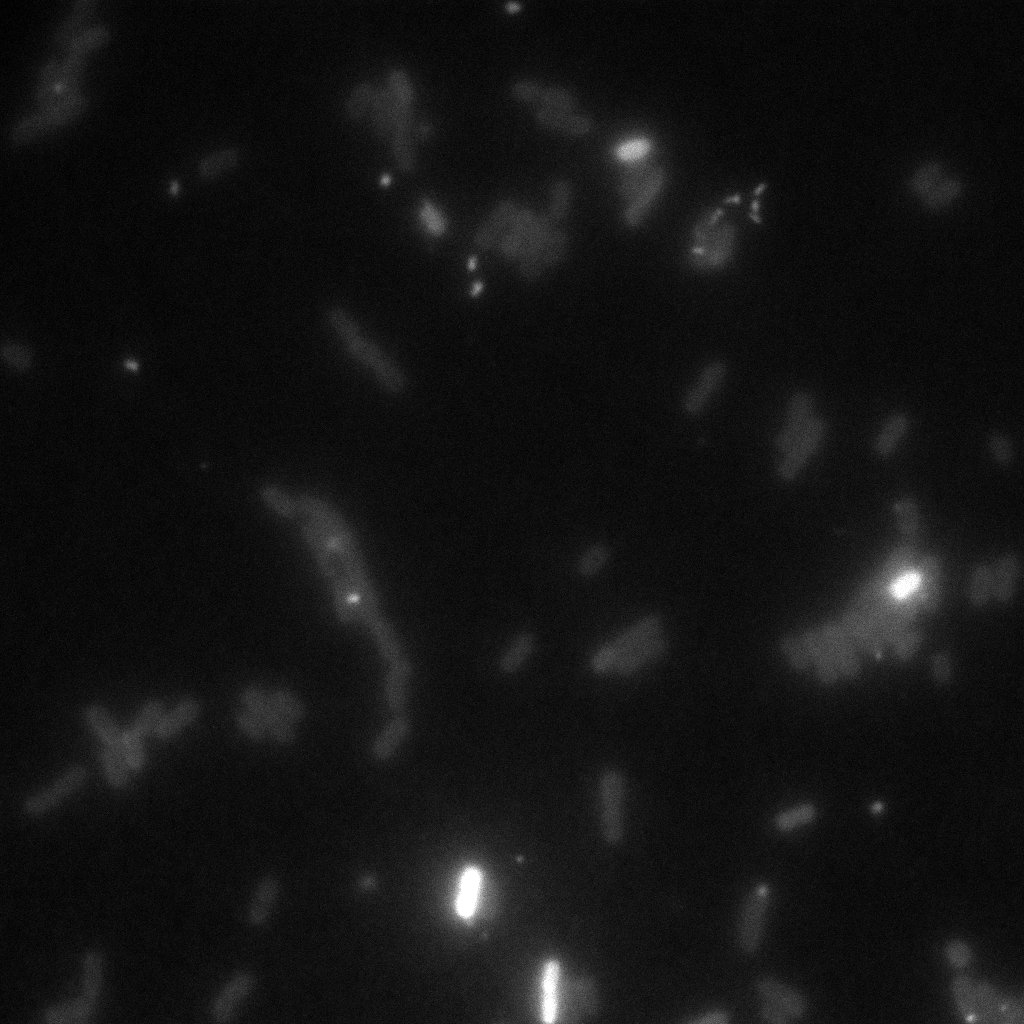

Supplement: Supplementary file 7 — Source data Fig. 3 [file 44321_2025_331_MOESM7_ESM.zip › FIGURE 3/3C/TIFF/10 min Untreated.tif]

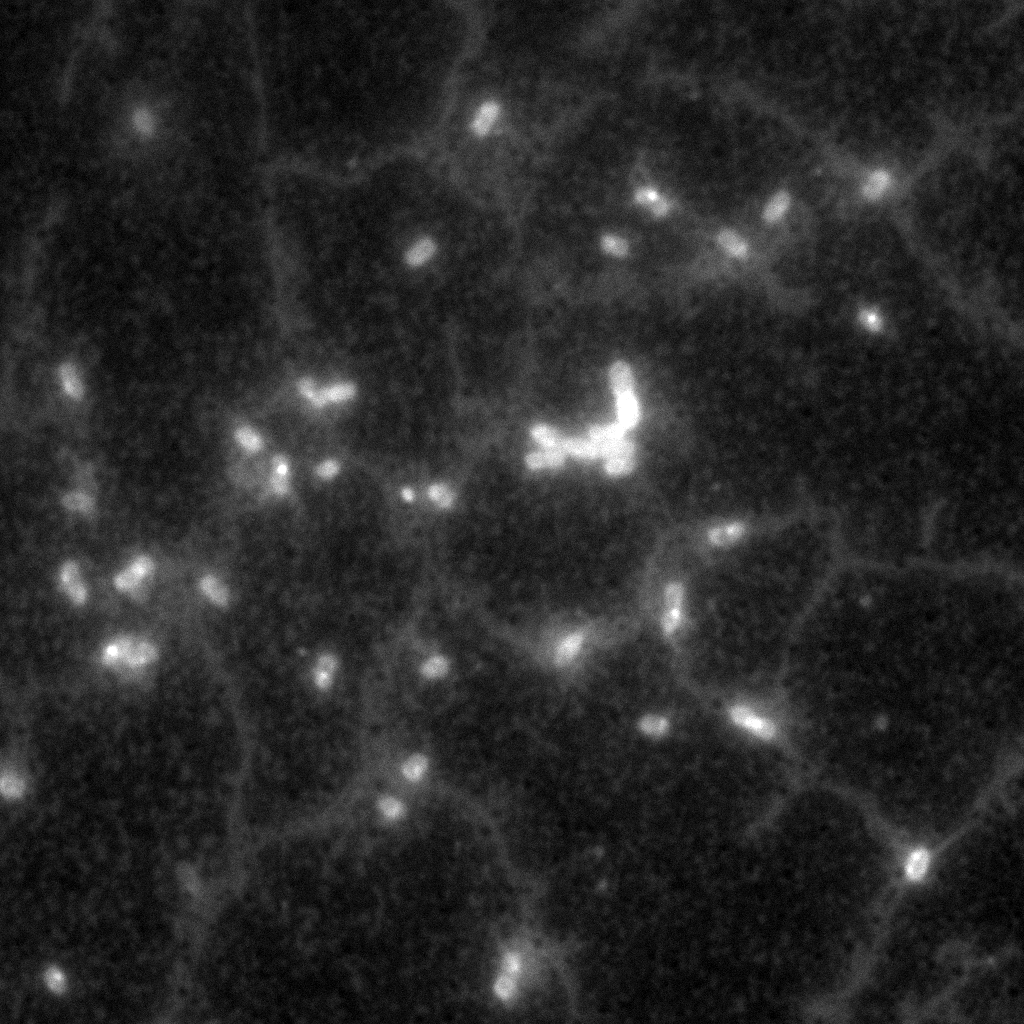

Supplement: Supplementary file 7 — Source data Fig. 3 [file 44321_2025_331_MOESM7_ESM.zip › FIGURE 3/3C/TIFF/10 min ENOblock 16 ug.tif]

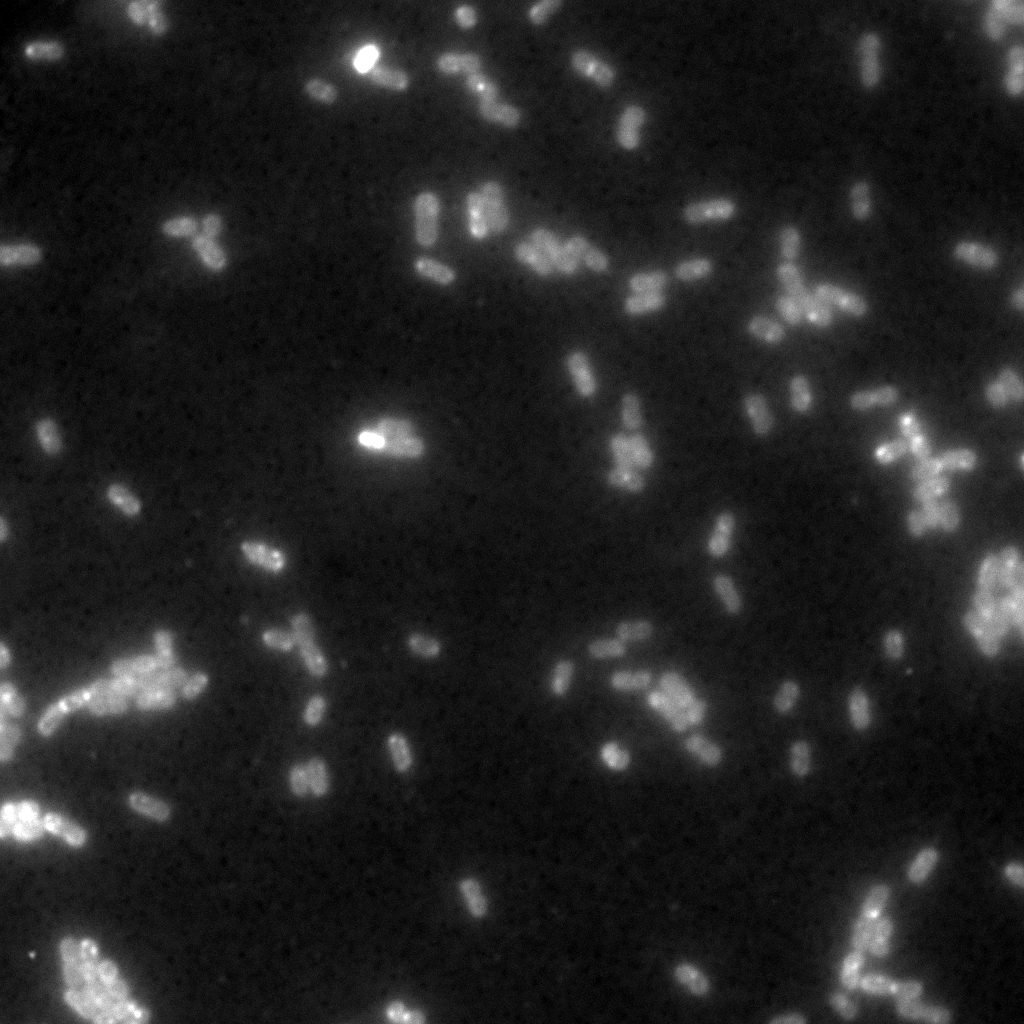

Supplement: Supplementary file 7 — Source data Fig. 3 [file 44321_2025_331_MOESM7_ESM.zip › FIGURE 3/3C/TIFF/10 min Enoblock 32 ug.tif]

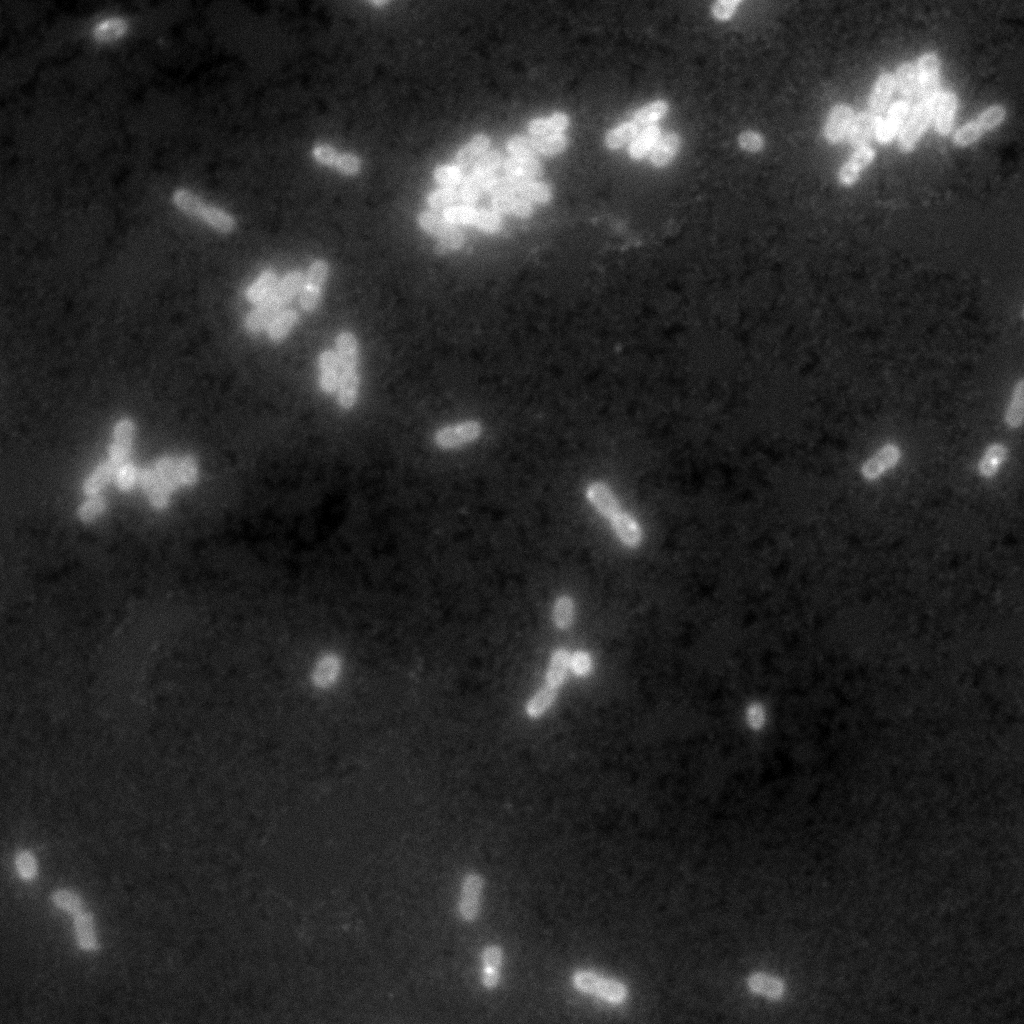

Supplement: Supplementary file 7 — Source data Fig. 3 [file 44321_2025_331_MOESM7_ESM.zip › FIGURE 3/3C/TIFF/10 min Colistin.tif]

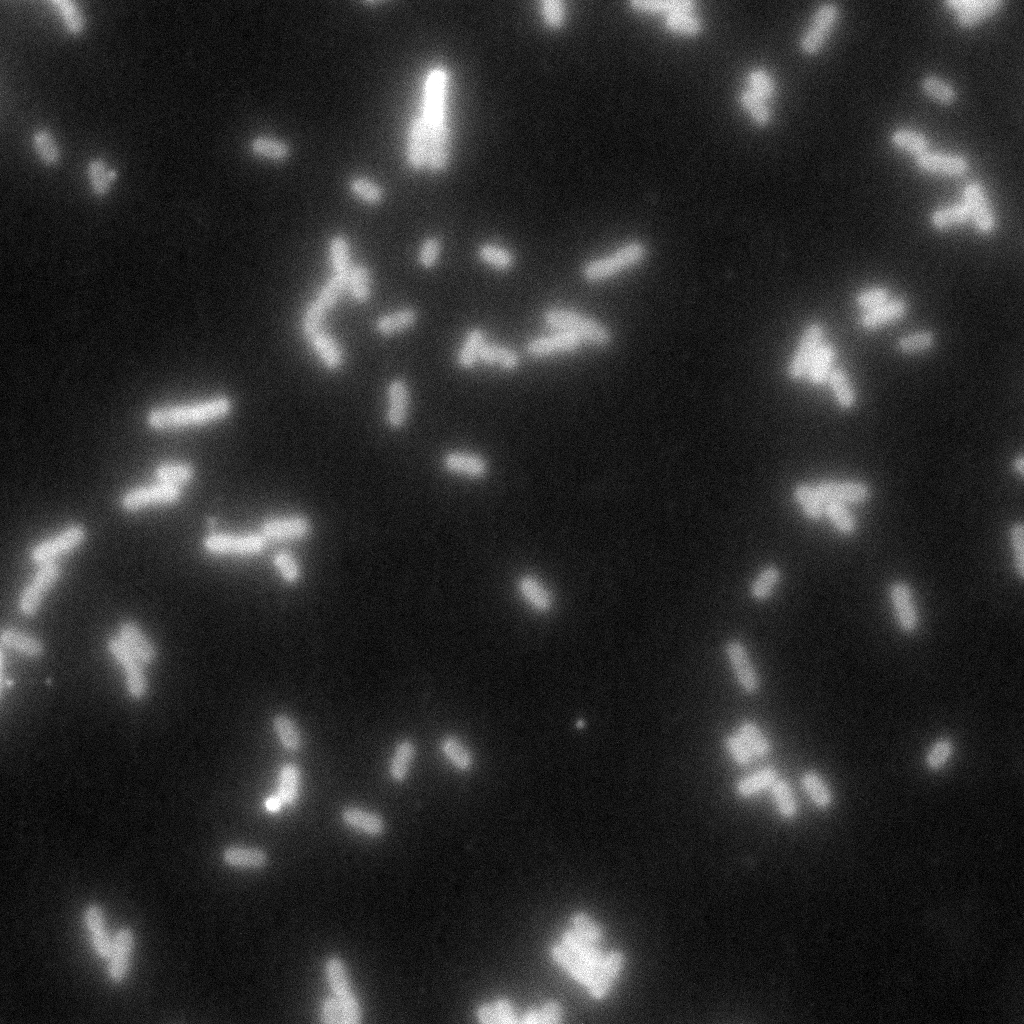

Supplement: Supplementary file 7 — Source data Fig. 3 [file 44321_2025_331_MOESM7_ESM.zip › FIGURE 3/3C/TIFF/60 min ENOblock 8 ug.tif]

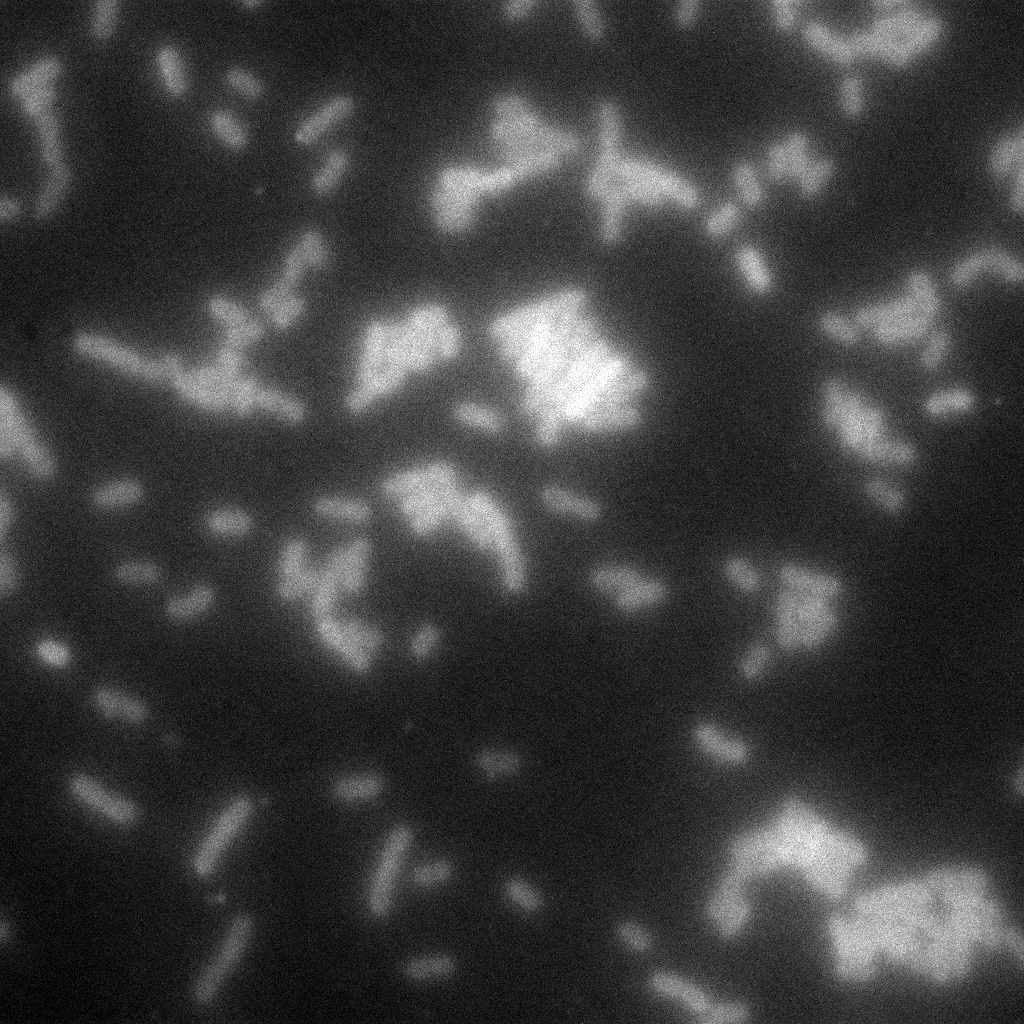

Supplement: Supplementary file 7 — Source data Fig. 3 [file 44321_2025_331_MOESM7_ESM.zip › FIGURE 3/3C/TIFF/30 min Untreated.tif]

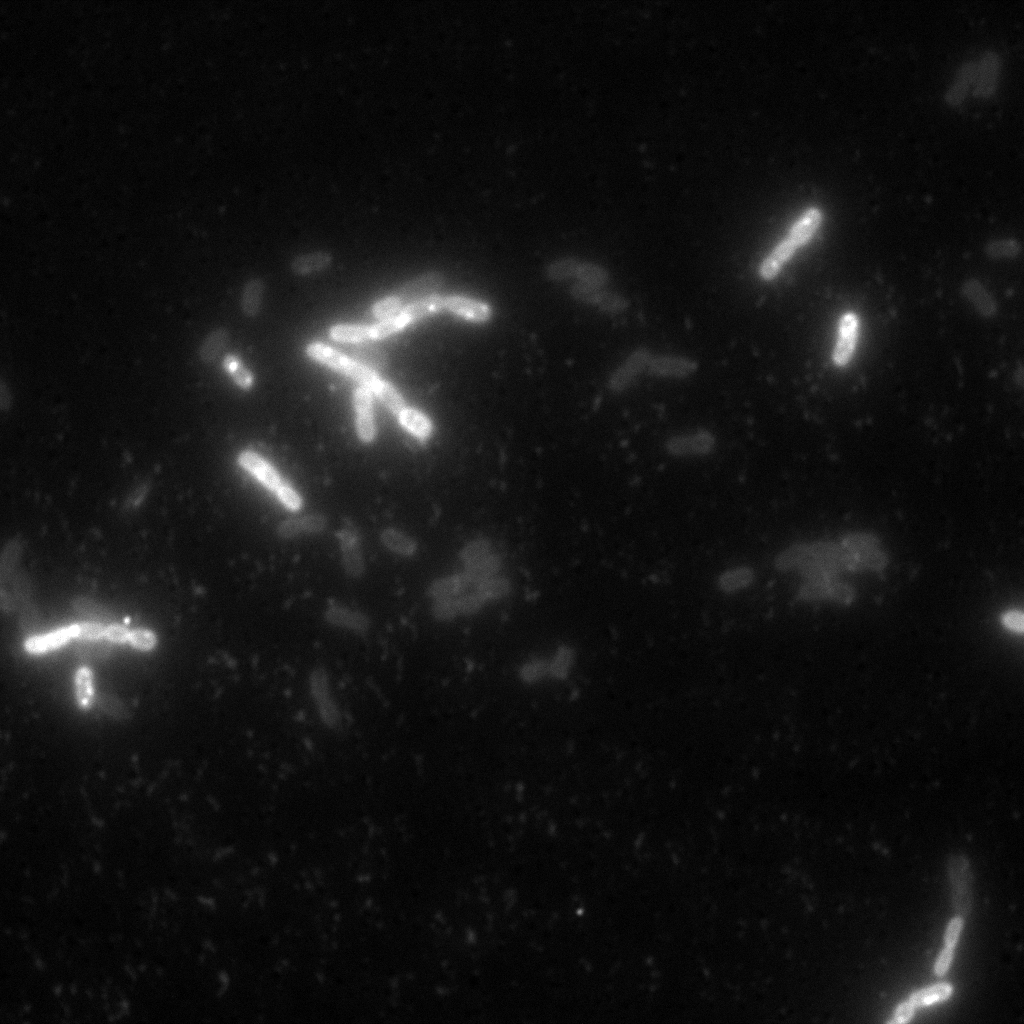

Supplement: Supplementary file 7 — Source data Fig. 3 [file 44321_2025_331_MOESM7_ESM.zip › FIGURE 3/3C/TIFF/10 min ENOblock 8 ug.tif]

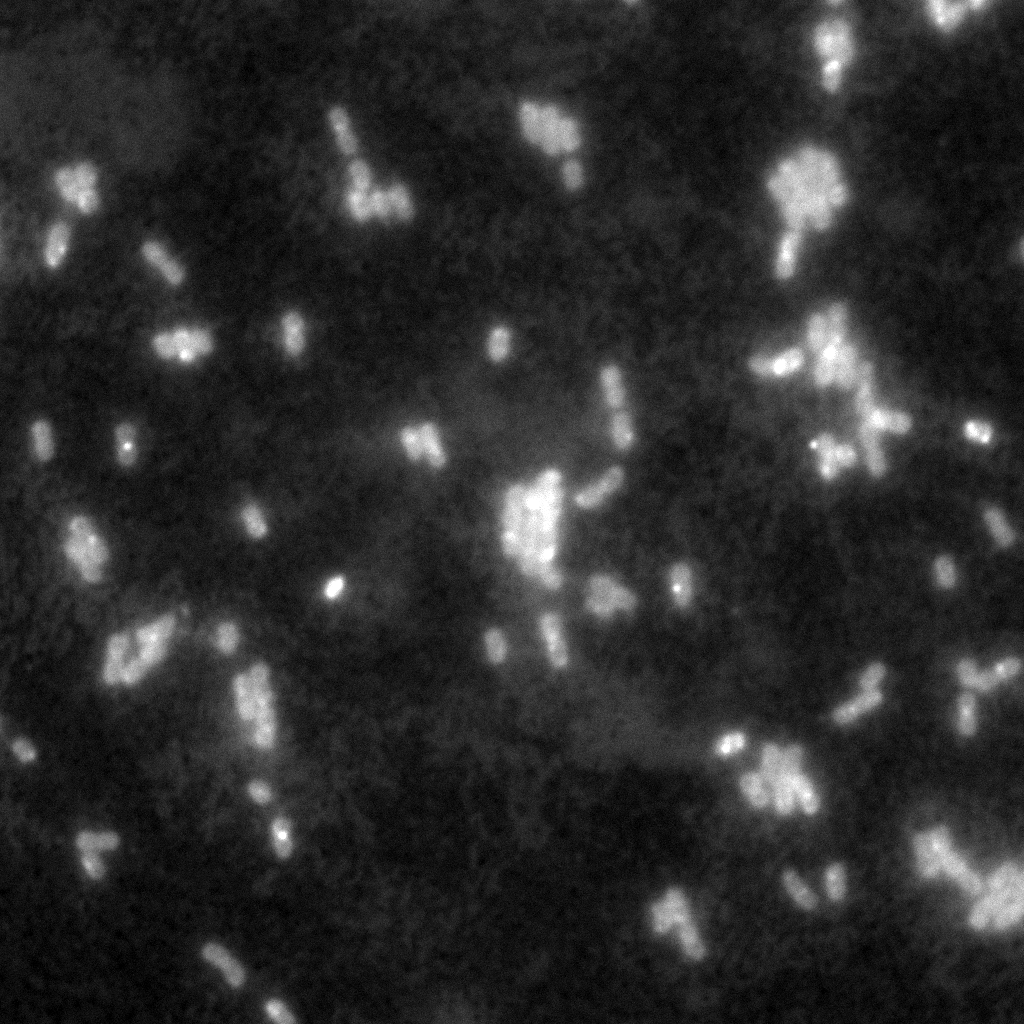

Supplement: Supplementary file 7 — Source data Fig. 3 [file 44321_2025_331_MOESM7_ESM.zip › FIGURE 3/3C/TIFF/30 min Enoblock 32 ug.tif]

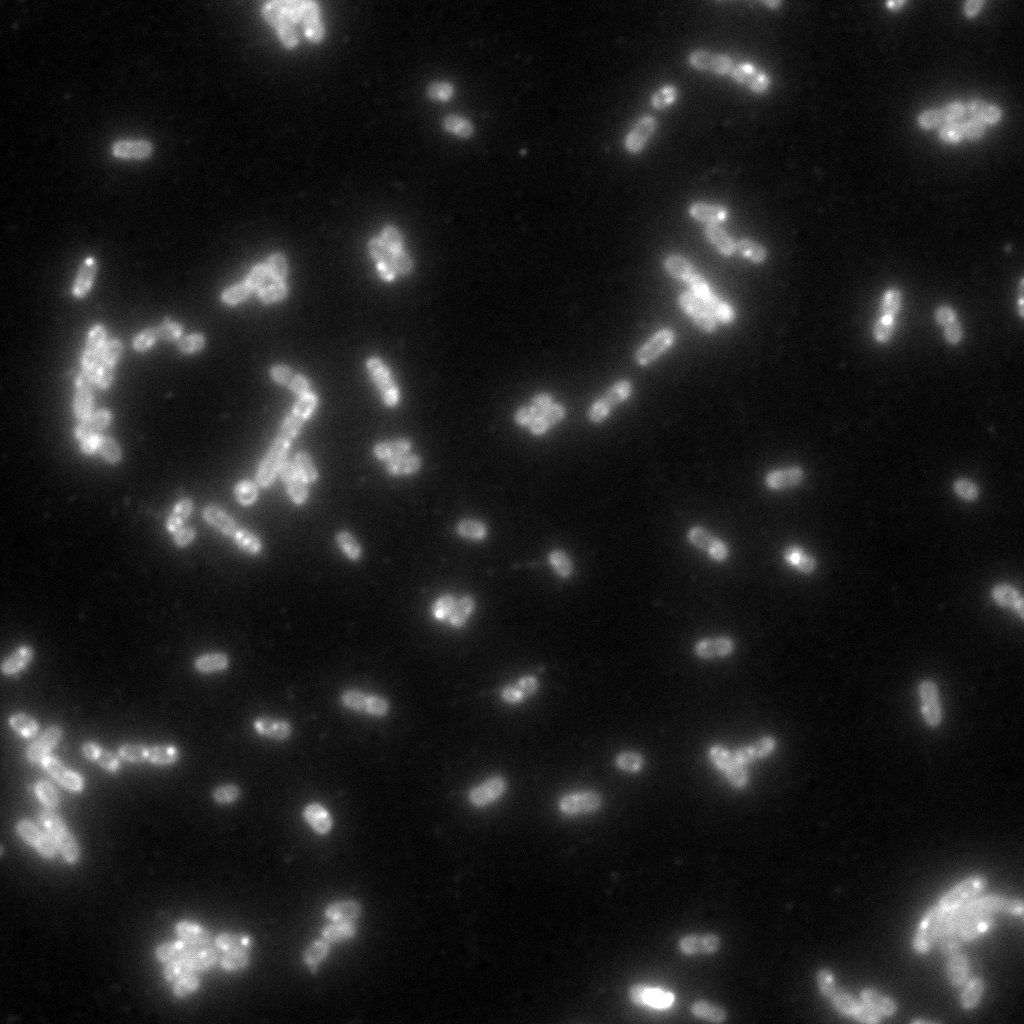

Supplement: Supplementary file 7 — Source data Fig. 3 [file 44321_2025_331_MOESM7_ESM.zip › FIGURE 3/3C/TIFF/30 min ENOblock 16 ug.tif]

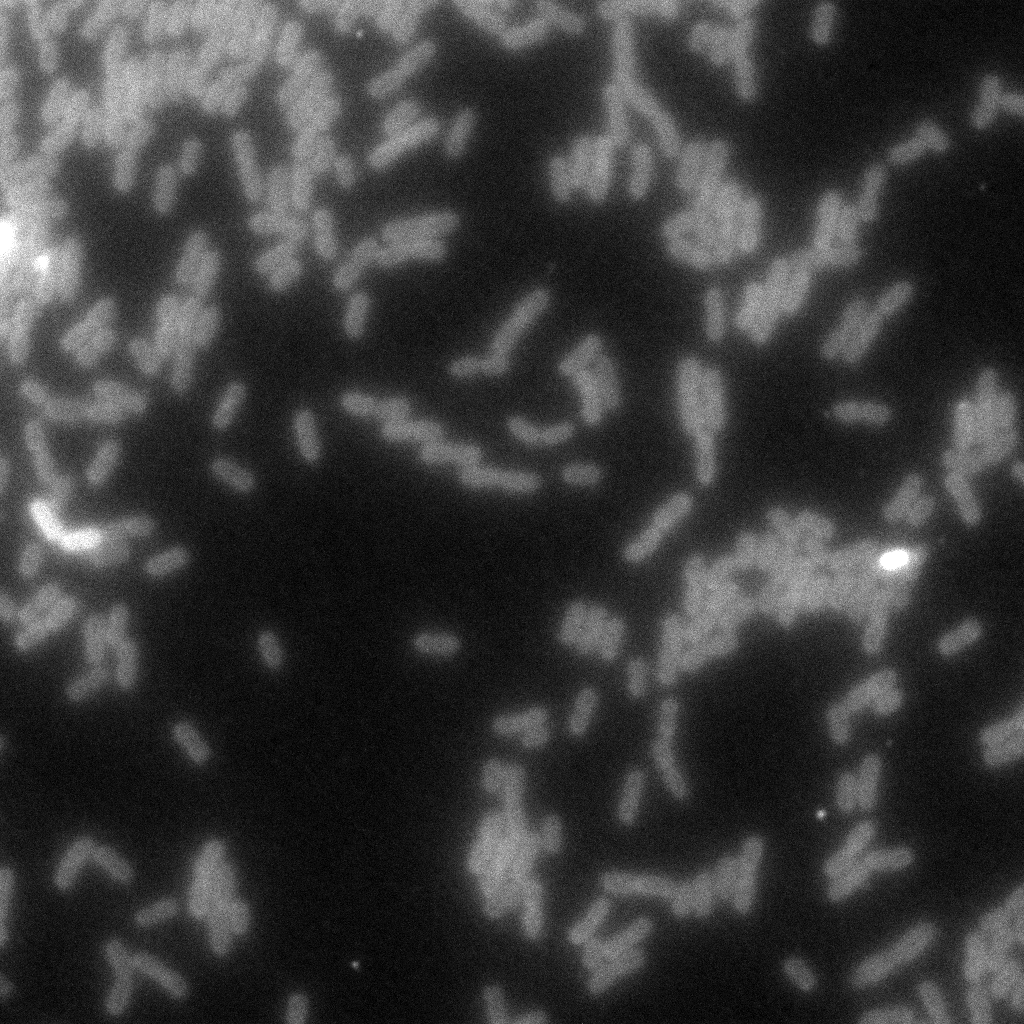

Supplement: Supplementary file 7 — Source data Fig. 3 [file 44321_2025_331_MOESM7_ESM.zip › FIGURE 3/3C/TIFF/60 min Untreated.tif]

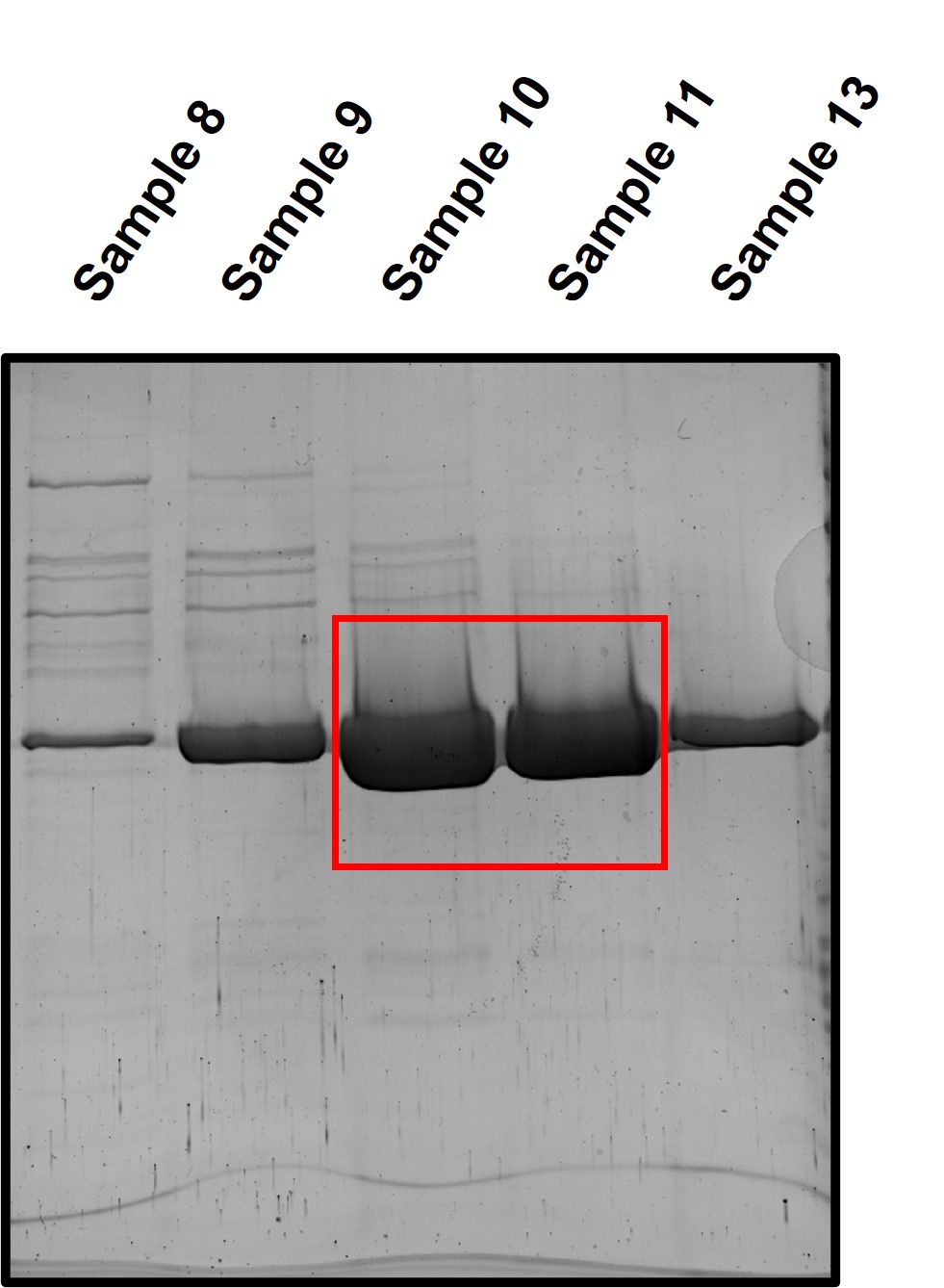

Supplement: Supplementary file 8 — Source data Fig. 4 [file 44321_2025_331_MOESM8_ESM.zip › FIGURE 4/4B/SDS-PAGE Enolase.jpg]

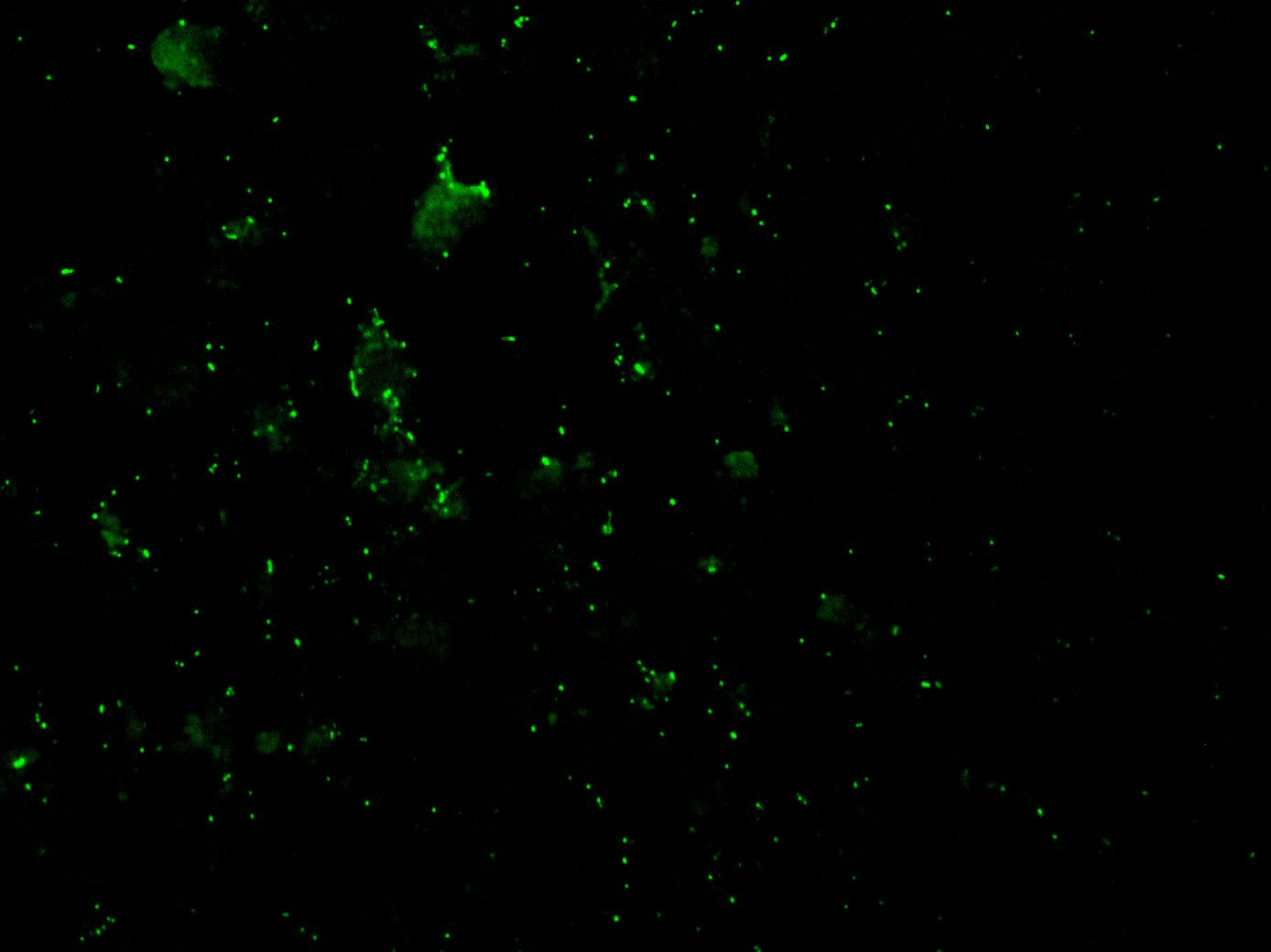

Supplement: Supplementary file 9 — Source data Fig. 5 [file 44321_2025_331_MOESM9_ESM.zip › FIGURE 5/5D/Ab CR17 Untreated anti-OmpA.tif]

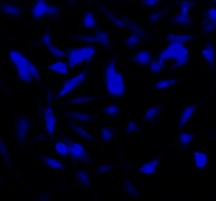

Supplement: Supplementary file 9 — Source data Fig. 5 [file 44321_2025_331_MOESM9_ESM.zip › FIGURE 5/5D/Ab ATCC 17978 ENOblock DAPI.tif]

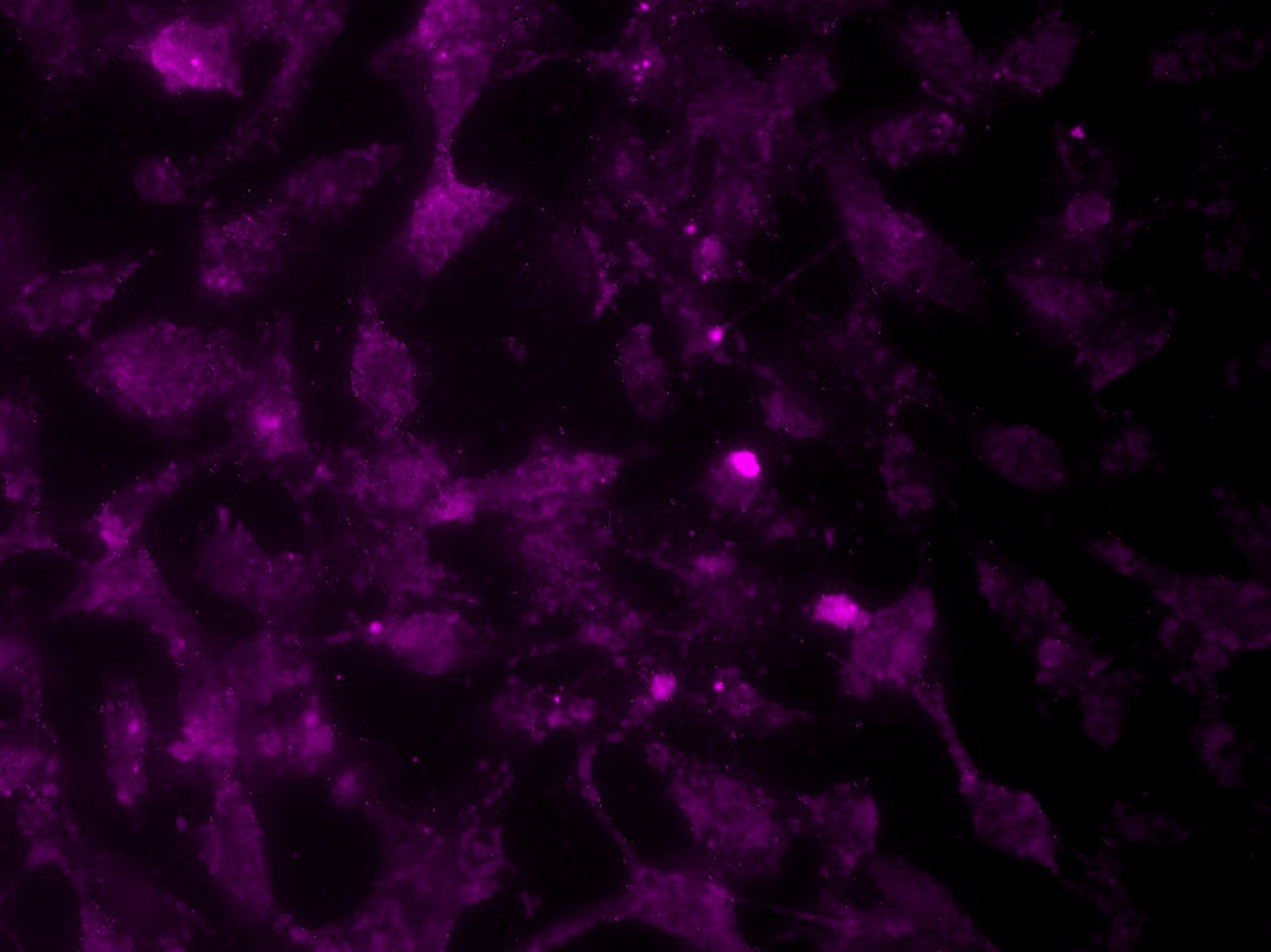

Supplement: Supplementary file 9 — Source data Fig. 5 [file 44321_2025_331_MOESM9_ESM.zip › FIGURE 5/5D/Ab CR17 Untreated anti-fibronectin.tif]

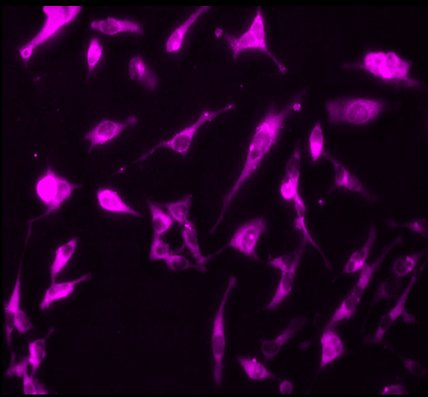

Supplement: Supplementary file 9 — Source data Fig. 5 [file 44321_2025_331_MOESM9_ESM.zip › FIGURE 5/5D/Ab CR17 ENOblock anti-fibronectin.tif]

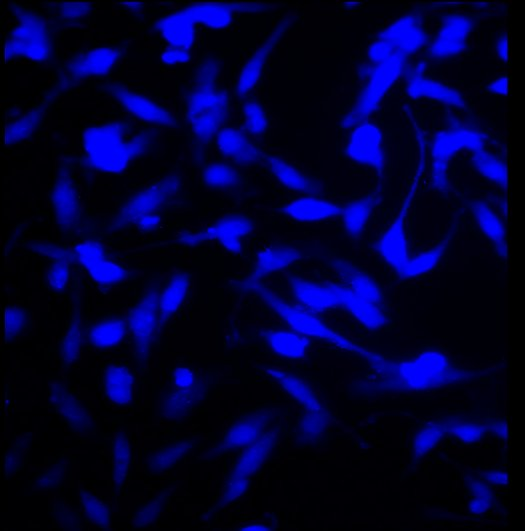

Supplement: Supplementary file 9 — Source data Fig. 5 [file 44321_2025_331_MOESM9_ESM.zip › FIGURE 5/5D/Ab ATCC 17978 Untreated DAPI.tif]

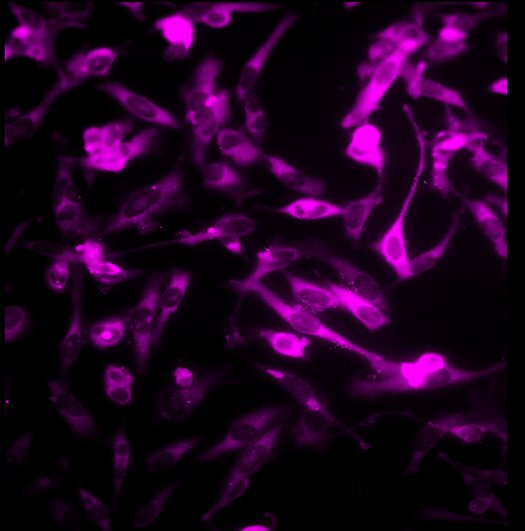

Supplement: Supplementary file 9 — Source data Fig. 5 [file 44321_2025_331_MOESM9_ESM.zip › FIGURE 5/5D/Ab ATCC 17978 Untreated anti-fibronectin.tif]

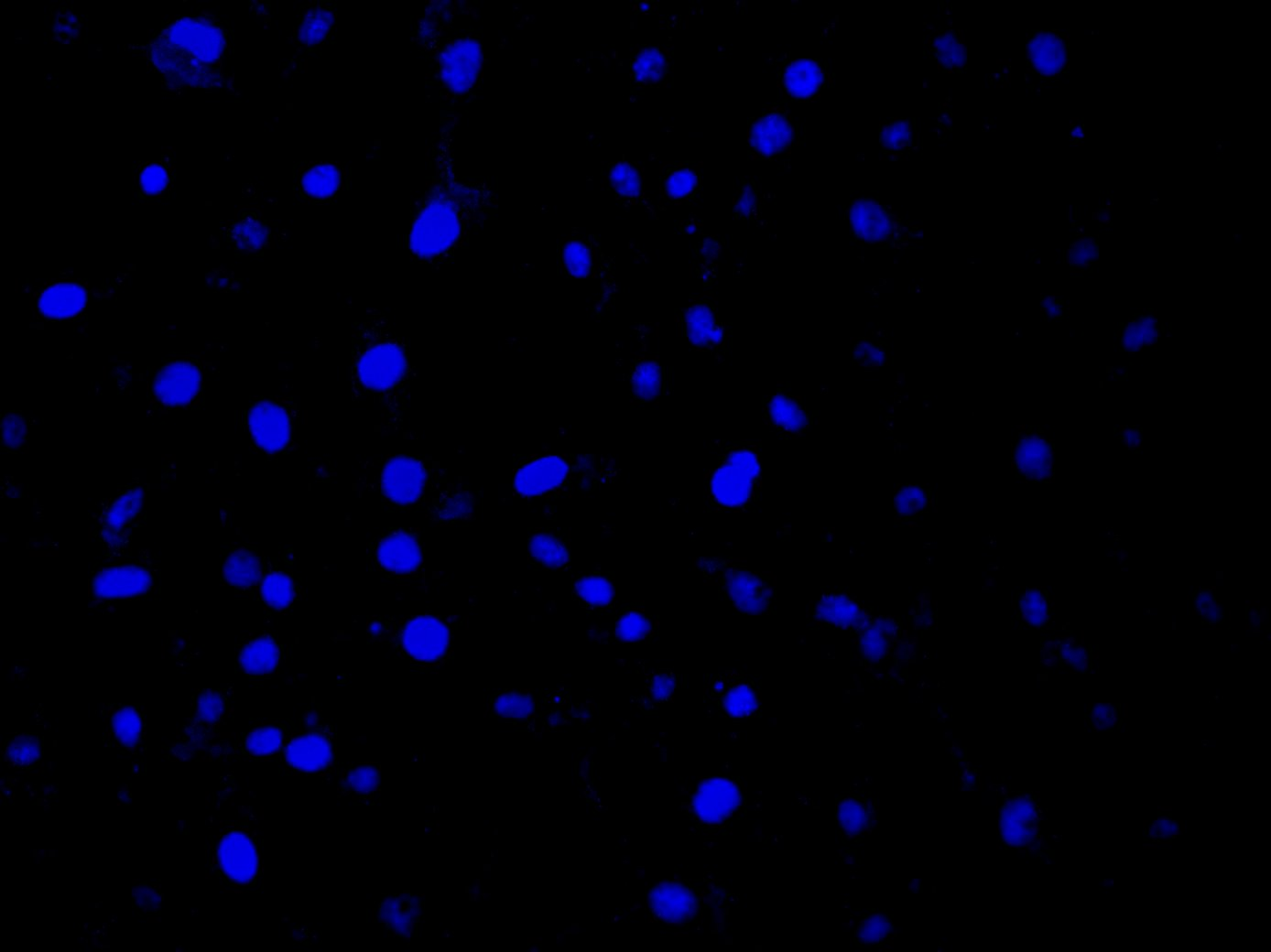

Supplement: Supplementary file 9 — Source data Fig. 5 [file 44321_2025_331_MOESM9_ESM.zip › FIGURE 5/5D/Ab CR17 Untreated DAPI.tif]

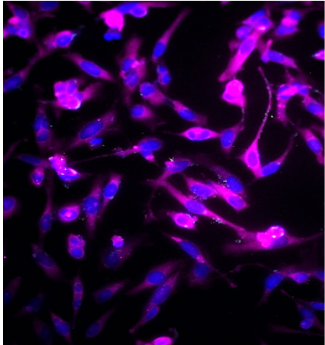

Supplement: Supplementary file 9 — Source data Fig. 5 [file 44321_2025_331_MOESM9_ESM.zip › FIGURE 5/5D/Ab ATCC 17978 merge.tif]

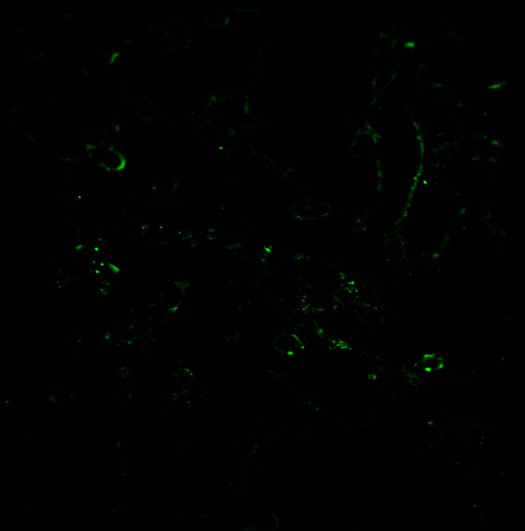

Supplement: Supplementary file 9 — Source data Fig. 5 [file 44321_2025_331_MOESM9_ESM.zip › FIGURE 5/5D/Ab ATCC 17978 Untreated anti-OmpA.tif]

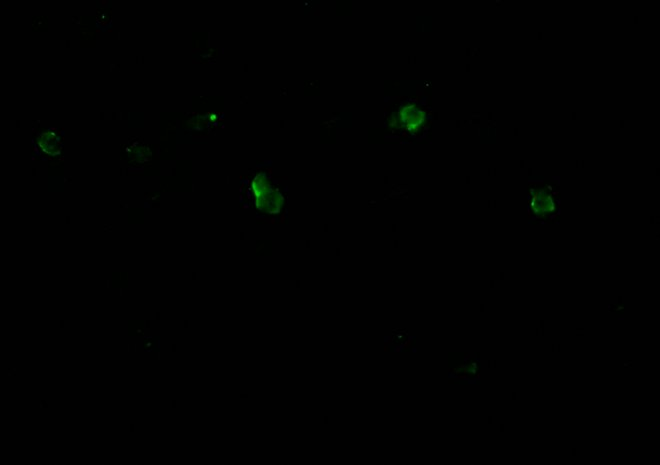

Supplement: Supplementary file 9 — Source data Fig. 5 [file 44321_2025_331_MOESM9_ESM.zip › FIGURE 5/5D/Ab ATCC 17978 ENOblock anti-OmpA.tif]

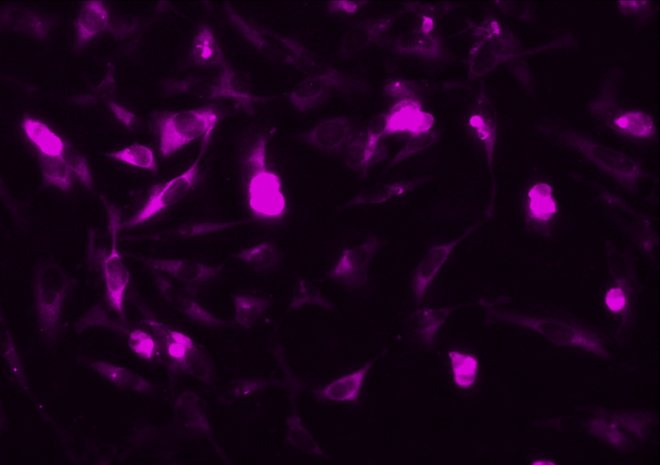

Supplement: Supplementary file 9 — Source data Fig. 5 [file 44321_2025_331_MOESM9_ESM.zip › FIGURE 5/5D/Ab ATCC 17978 ENOblock anti-fibronectin.tif]

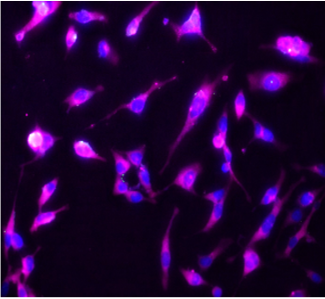

Supplement: Supplementary file 9 — Source data Fig. 5 [file 44321_2025_331_MOESM9_ESM.zip › FIGURE 5/5D/Ab CR17 ENOblock merge.tif]

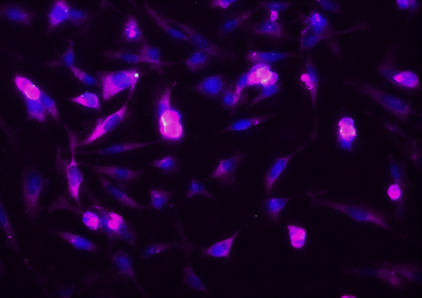

Supplement: Supplementary file 9 — Source data Fig. 5 [file 44321_2025_331_MOESM9_ESM.zip › FIGURE 5/5D/Ab ATCC 17978 ENOblock merge.tif]

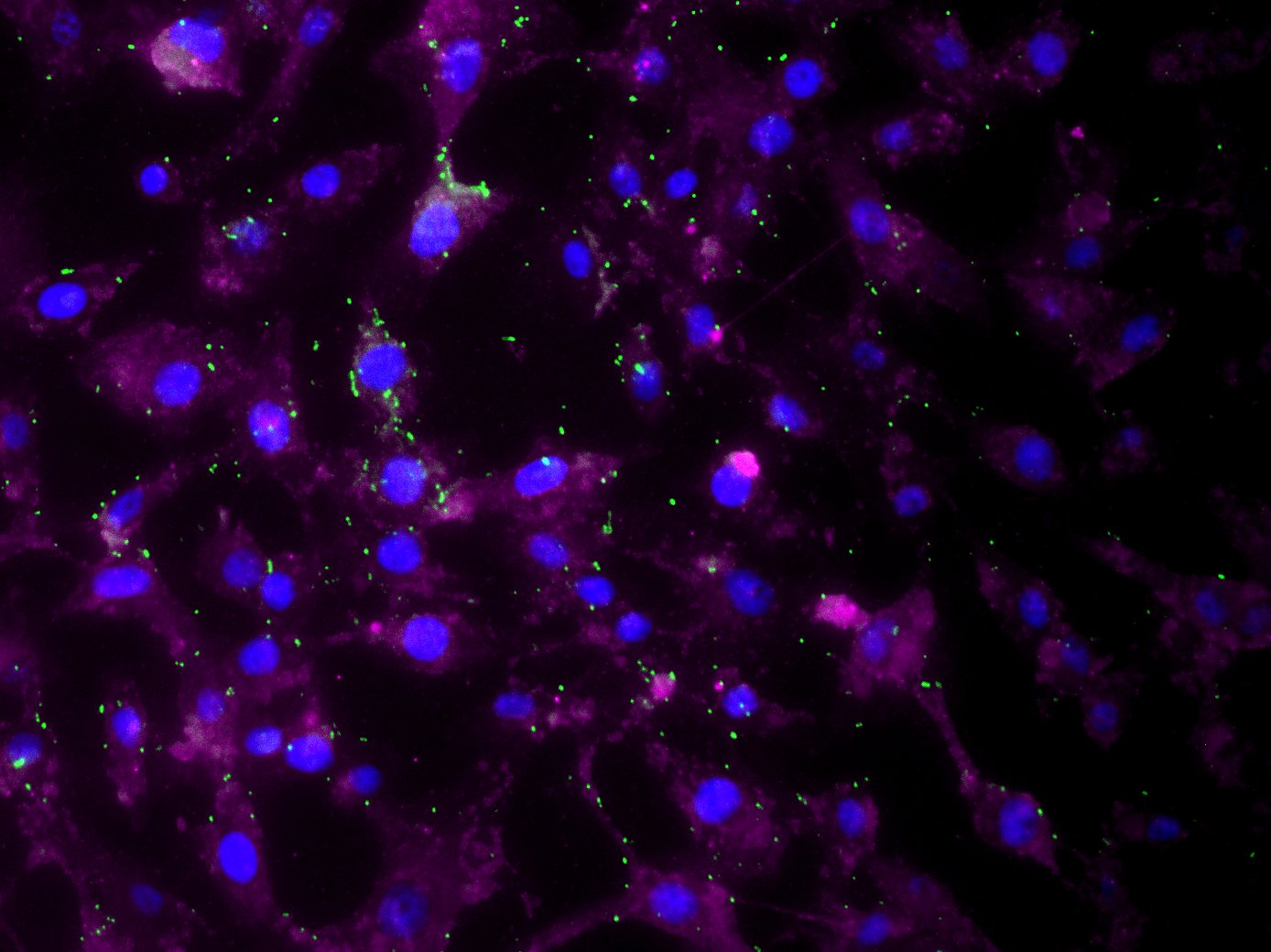

Supplement: Supplementary file 9 — Source data Fig. 5 [file 44321_2025_331_MOESM9_ESM.zip › FIGURE 5/5D/Ab CR17 merge.tif]

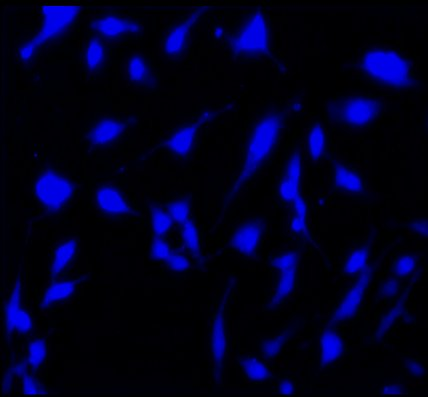

Supplement: Supplementary file 9 — Source data Fig. 5 [file 44321_2025_331_MOESM9_ESM.zip › FIGURE 5/5D/Ab CR17 ENOblock DAPI.tif]

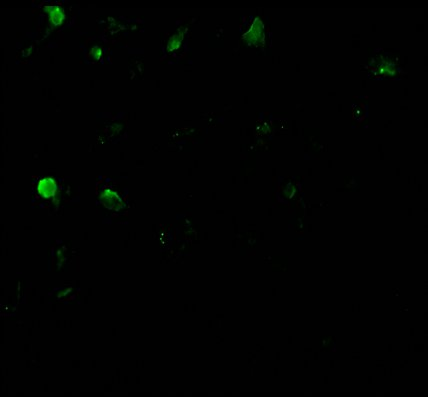

Supplement: Supplementary file 9 — Source data Fig. 5 [file 44321_2025_331_MOESM9_ESM.zip › FIGURE 5/5D/Ab CR17 ENOblock anti-OmpA.tif]

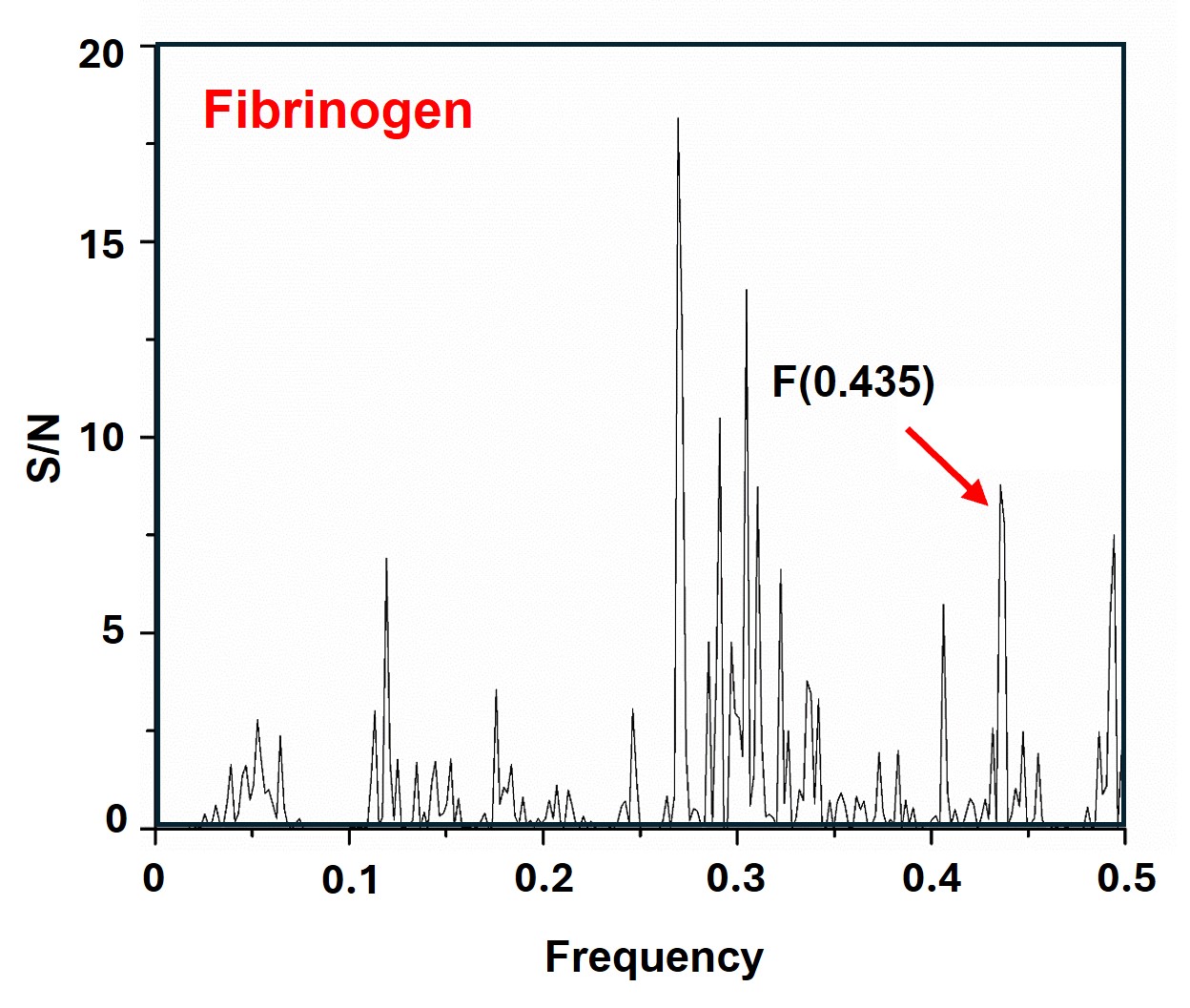

Supplement: Supplementary file 11 — Source data Fig. 7 [file 44321_2025_331_MOESM11_ESM.zip › FIGURE 7/7A-C/ISM Fibrinogen enolase ENOblock.jpg]

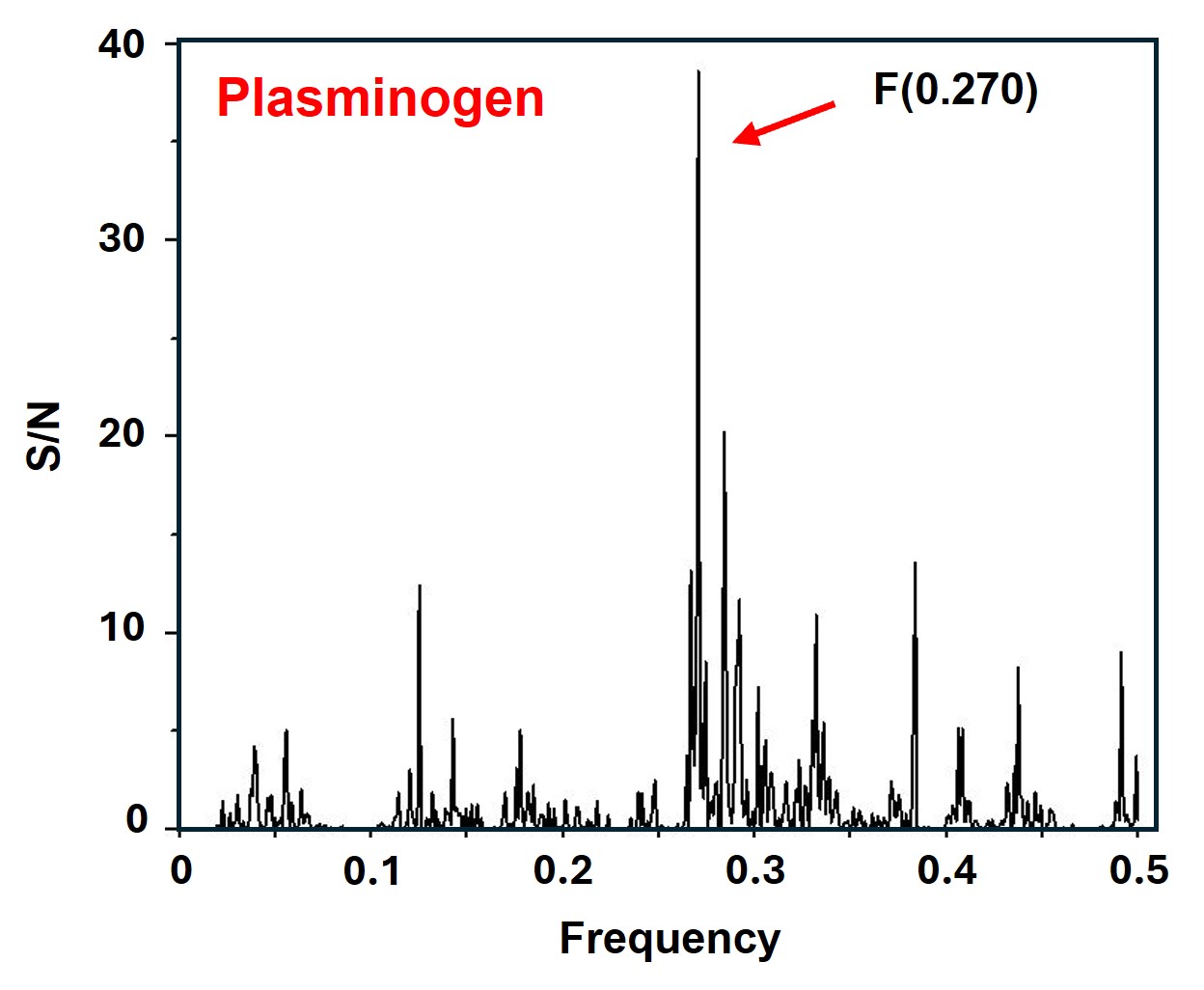

Supplement: Supplementary file 11 — Source data Fig. 7 [file 44321_2025_331_MOESM11_ESM.zip › FIGURE 7/7A-C/ISM Plasminogen enolase ENOblock.jpg]

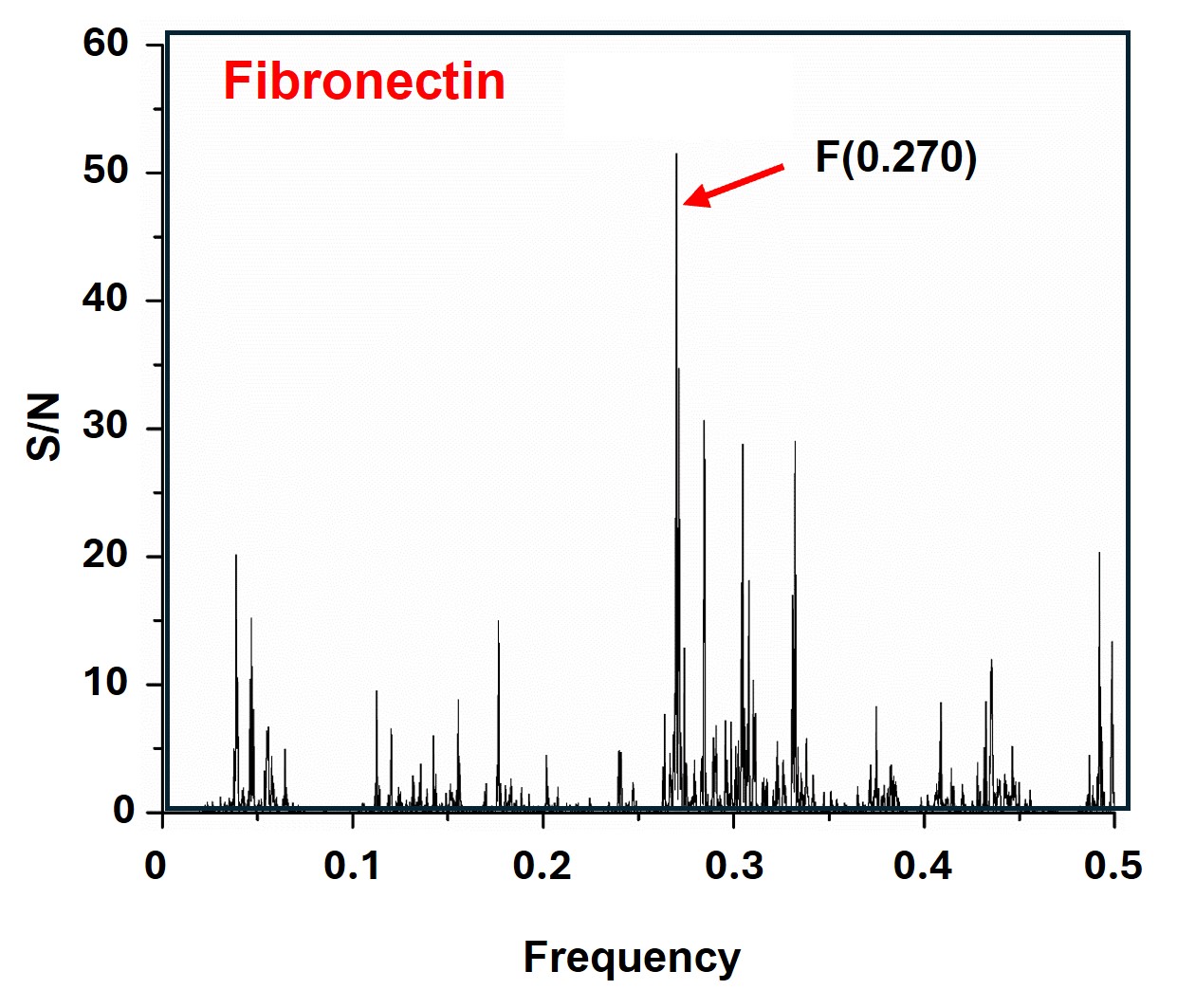

Supplement: Supplementary file 11 — Source data Fig. 7 [file 44321_2025_331_MOESM11_ESM.zip › FIGURE 7/7A-C/ISM Fibronectin enolase ENOblock.jpg]
