# Supplementary material for: ENOblock synergizes with colistin to treat Acinetobacter baumannii infections
Source: EMBO Mol Med. 2025 Oct 31;17(12):3496–524. doi: 10.1038/s44321-025-00331-2 (PMC12686454; doi:10.1038/s44321-025-00331-2)
Supplement: Supplementary file 11 — Source data Fig. 7 [file 44321_2025_331_MOESM11_ESM.zip › FIGURE 7/7A-C/ISM frequencies for ENOblock intermolecular interactions and corresponding signal ΓÇô to ΓÇô noise (S-N) ratios..docx]

| **Interaction** | **F** | **S/N** |
| --- | --- | --- |
| Enolase - ENOblock | 0.271 | 12.44 |
|  | 0.435 | 3.40 |
| Fibronectin - enolase - ENOblock | 0.271 | 51.498 |
|  | 0.435 | 11.96 |
| Fibrinogen - enolase - ENOblock | 0.271 | 18.161 |
|  | 0.435 | 8.78 |
