# Supplementary material for: ENOblock synergizes with colistin to treat Acinetobacter baumannii infections
Source: EMBO Mol Med. 2025 Oct 31;17(12):3496–524. doi: 10.1038/s44321-025-00331-2 (PMC12686454; doi:10.1038/s44321-025-00331-2)
Supplement: Supplementary file 13 — Expanded View Figures [file 44321_2025_331_MOESM13_ESM.pdf]

Expanded View Figures

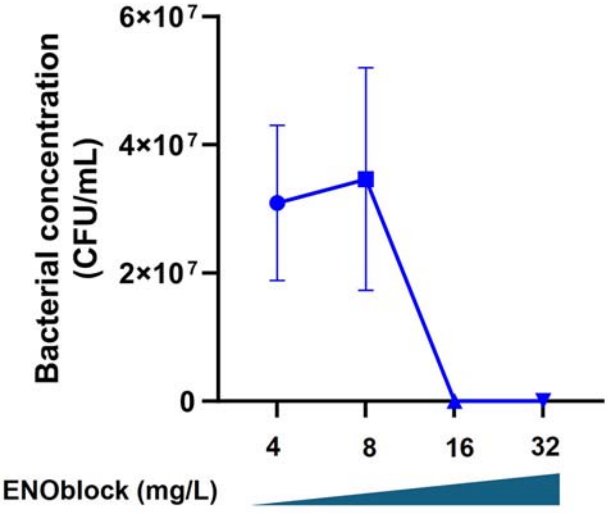

Figure EV1. ENOblock selective pressure.

A. baumannii Ab ATCC 17978 concentrations after incubation with increasing concentrations of ENOblock.

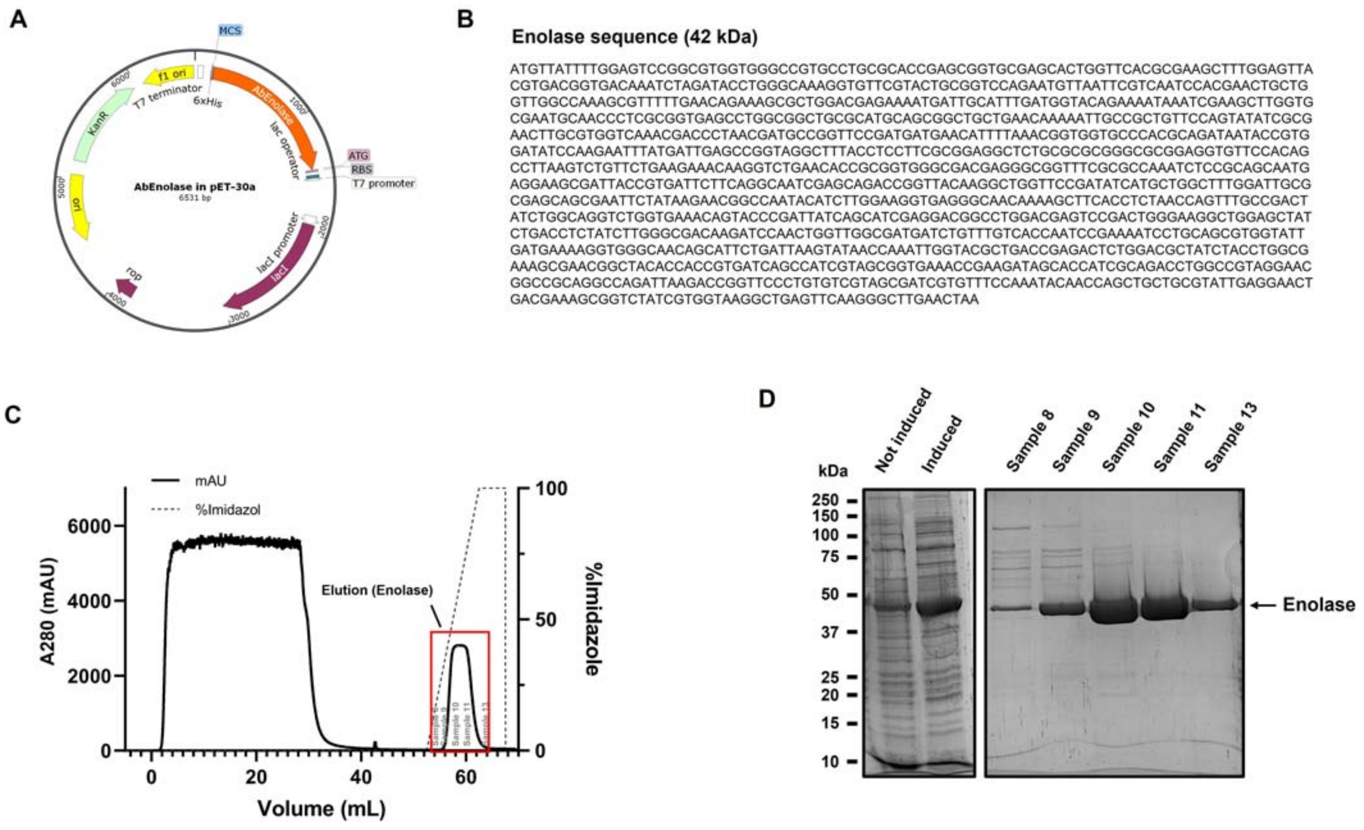

Figure EV2. Construction and characterization of *A.baumannii* enolase.

(**A**) Illustration of the recombinant expression vector of enolase. (**B**) The sequence of the enolase. (**C**) Purification of enolase using the Histrap FF column. (**D**) SDS-PAGE gel analysis of the purified enolase.

© The Author(s)

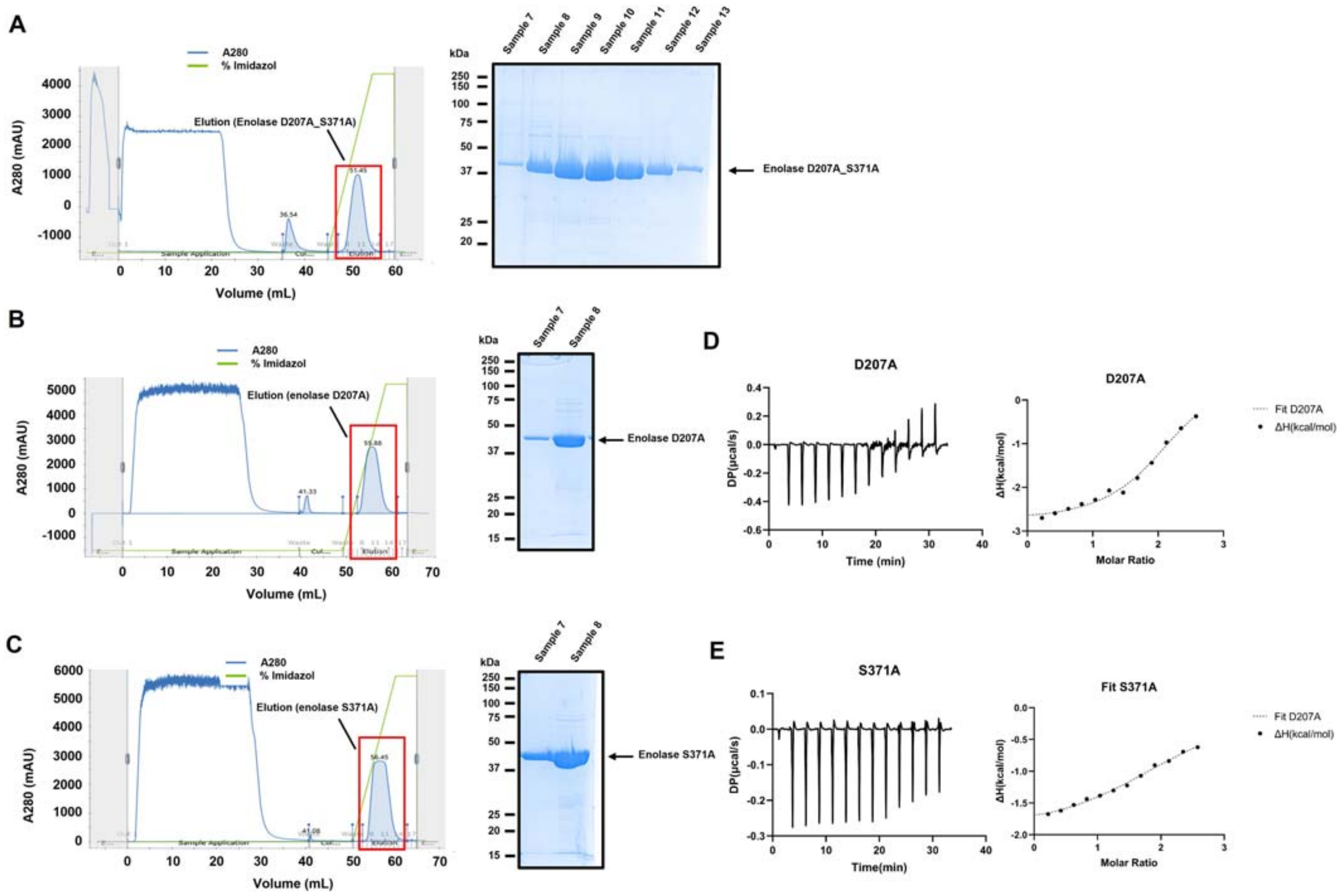

Figure EV3. Construction and characterization of *A. baumannii* enolase mutants.

(**A**–**C**) Purification and SDS-PAGE gel analysis of the purified enolase D207_S371, enolase D207A and enolase S371 using the Histrap FF column. (**E**, **F**) Isothermal titration calorimetry (ITC) titrations with integrated fitted heat plots of ENOblock binding with enolase (D207A) or enolase (S371A).

Q14

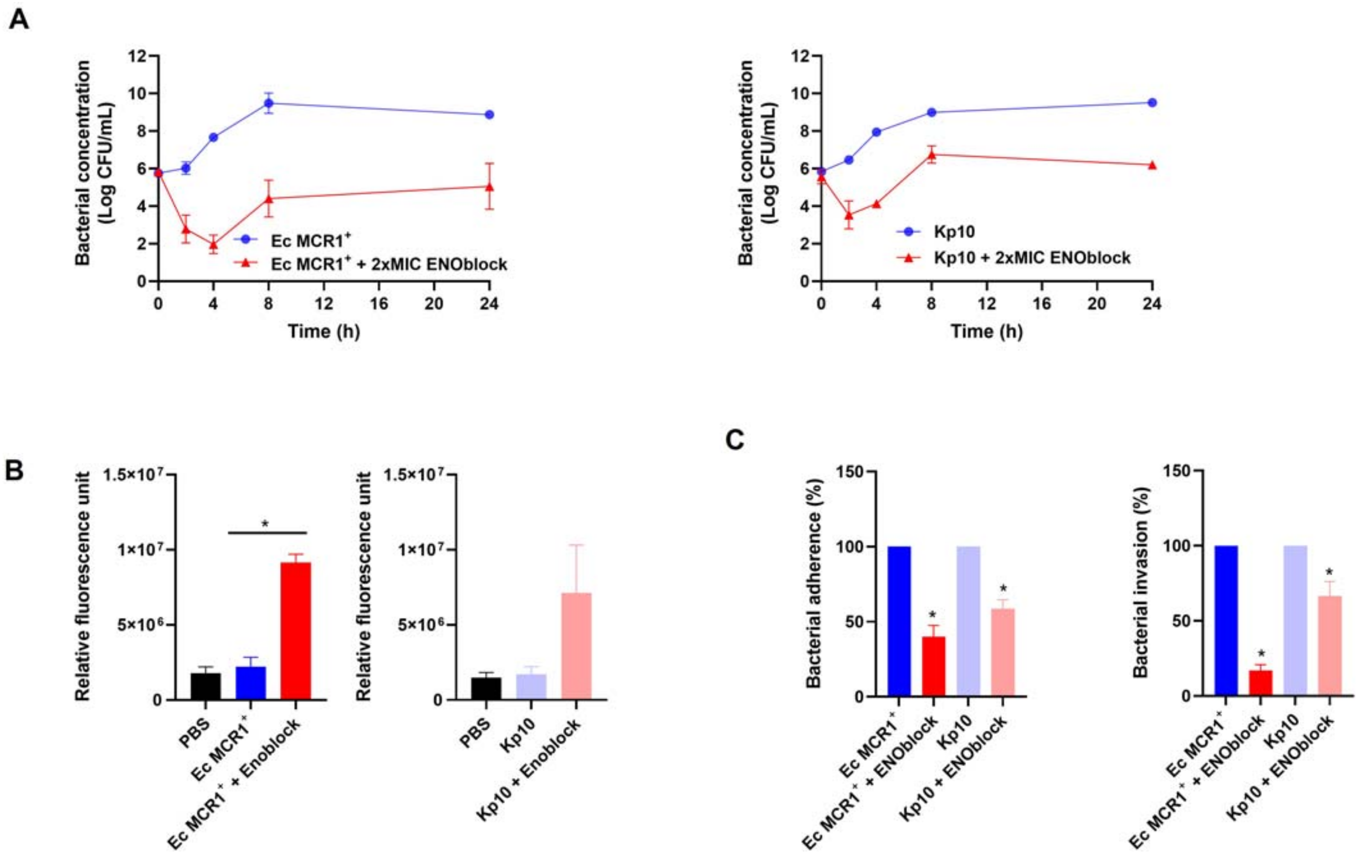

Figure EV4. ENOblock is active against *E. coli and K. pneumoniae.*

Q15

(**A**) Time-kill curves of *E. coli* Ec MCR1$^+$ and Kp10 strains in the presence of 2xMIC ENOblock for 24 h. (**B**) Membrane permeabilization of *E. coli* Ec MCR1$^+$ and Kp10 strains in the presence of 0.5xMIC ENOblock, incubated for 10 min, was quantified by Typhon Scanner. Data are represented as mean ± SEM from three independent replicates and experiments. **P* = 0.001: Ec MCR1$^+$ vs Ec MCR1$^+$ + ENOblock(two-tailed Student's *t* test). (**C**) Analysis of *E. coli* Ec MCR1$^+$ and Kp10 strains adhesion into HeLa cells with (1xMIC) and without ENOblock treatment. The data are presented as means ± SEM, **P* = 0.015: Ec MCR1$^+$ vs Ec MCR1$^+$ + ENOblock and **P* = 0.02: Kp10 vs Kp10 + ENOblock (two-tailed Student's *t* test). (**D**) Analysis of *E. coli* Ec MCR1$^+$ and Kp10 strains invasion into HeLa cells with (1xMIC) and without ENOblock treatment. The data are presented as means ± SEM, **P* = 0.002: Ec MCR1$^+$ vs Ec MCR1$^+$ + ENOblock and **P* = 0.037: Kp10 vs Kp10 + ENOblock treatment (two-tailed Student's *t* test).

 © The Author(s)

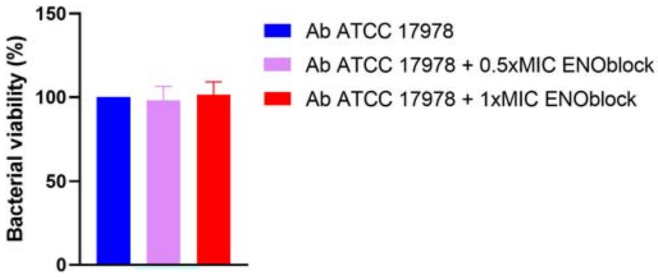

Figure EV5. Bacterial viability after incubation of ATCC with 0.5 and 1×MIC during 30 min with ENOblock.
